# Stress-induced helicase DHX36 controls neuroinflammation

**DOI:** 10.1016/j.jbc.2026.113584

**Published:** 2026-09-23

**Authors:** Vijay Kumar M J, Rocio Diaz Escarcega, Chunfeng Tan, Jose F. Moruno-Manchon, Akihiko Urayama, Sean P. Marrelli, Rodrigo Morales, Louise D. McCullough, Nayun Kim, Sung Yun Jung, David Monchaud, Andrey S. Tsvetkov

**Affiliations:** 1 Department of Neurology, The University of Texas McGovern Medical School, Houston, Texas, USA; 2 The BRAINS Research Laboratory, The Department of Neurology, University of Texas McGovern Medical School, Houston, Texas, USA; 3 Centro Integrativo de Biologia y Quimica Aplicada (CIBQA), Universidad Bernardo O’Higgins, Santiago, Chile; 4 The University of Texas Graduate School of Biomedical Sciences, Houston, Texas, USA; 5 Division of Pharmacology and Toxicology, College of Pharmacy, The University of Texas at Austin, Austin, Texas, USA; 6 Department of Biochemistry and Molecular Pharmacology, Baylor College of Medicine, Houston, Texas, USA; 7 Institut de Chimie Moléculaire (ICMUB), UBFC Dijon, CNRS UMR6302, 9, Dijon, France

**Keywords:** G-quadruplex, DHX36, aging, neurodegeneration, cellular stress, neuroinflammation, Alzheimer’s disease, Hmga1, Hmga2, chromatin remodeling

## Abstract

Neurons in the aging brain experience sustained inflammatory, proteostatic, and genotoxic stress, accompanied by the accumulation of non-canonical nucleic acid structures such as G-quadruplexes (G4s). Using a G4-binding probe and mass spectrometry, we identified the G4 helicase DHX36 enriched in the aged mouse brain, prompting further investigation into its regulation during aging. DHX36 expression was elevated in neurons from aged mouse and human brains and further increased in a tauopathy mouse model and Alzheimer’s disease brain. Unexpectedly, pharmacological stabilization of G4s with pyridostatin (PDS) reduced *Dhx36* mRNA and protein levels in cultured mouse cortical neurons, indicating that G4 stabilization alone is insufficient to induce DHX36. In contrast, aging-relevant stressors, including inflammatory, ER, and genotoxic stress, robustly increased DHX36 expression and nuclear G4-positive puncta in cultured neurons. Transcriptomic profiling in neurons revealed that DHX36 suppresses neuroinflammatory pathways and modulates genes associated with inflammation, senescence, and chromatin regulation. DHX36 reduced expression of the chromatin architectural factors *Hmga1* and *Hmga2*, identifying them as downstream targets. An ATPase-deficient DHX36 mutant (E335A) reproduced the major transcriptional effects of wild-type DHX36, indicating that they do not require ATP-dependent unwinding. These findings identify DHX36 as a stress-responsive G4 helicase that links aging-relevant stress to transcriptional regulation in neurons and suggest a role in maintaining neuronal homeostasis during aging and neurodegeneration.

Nucleic acids adopt a range of secondary structures beyond the canonical double helix, including G-quadruplexes (G4s), i-motifs, triplex DNA, and R-loops (1, 2, 3, 4, 5, 6, 7, 8, 9, 10, 11, 12, 13, 14). Among these, G4s are four-stranded structures formed in guanine-rich regions of DNA and RNA *via* Hoogsteen hydrogen bonding and G-quartet stacking (1, 15, 16). G4-forming sequences are widespread in the genome and transcriptome and are enriched in regulatory regions such as promoters, enhancers, telomeres, and untranslated regions, where they can influence transcription, chromatin organization, and RNA metabolism (6, 17, 18, 19, 20, 21, 22). Cellular G4 levels are dynamically controlled by G4-binding proteins and G4 helicases, which resolve or stabilize these structures in a context-dependent manner (7, 23, 24, 25, 26). Although G4 structures have been studied extensively in cancer, much less is known about how G4s are regulated in the aging brain. Given that G4 homeostasis depends on helicases, their regulation in aging is of particular interest.

G4 helicases are closely linked to aging processes. Mutations in helicases such as WRN, BLM, FANCJ, RTEL1, and RECQL4 cause premature aging syndromes characterized by chromosomal instability, premature cellular senescence, and accelerated organismal aging, underscoring the need for tight G4 regulation (27, 28, 29, 30, 31). Many of these proteins function as regulators of transcription and signaling. For example, DDX5 and DDX17 recruit transcriptional regulators to gene promoters (32, 33). RECQL4 scaffolds replication factors (34). WRN stimulates FEN1 endonuclease activity through direct interaction (35). In innate immunity, DHX36 forms a complex with DDX1 and DDX21 to sense cytosolic double-stranded RNA (dsRNA) and activate TRIF-dependent signaling (36). Importantly, several of these helicases carry out functions that do not require their ATPase or unwinding activity. RECQL5 binds RNA polymerase II and directly inhibits transcription, and an ATPase-deficient RECQL5 mutant retains this activity (37, 38). DDX5 and DDX17 regulate transcription through interactions with transcriptional regulators, including HDAC1 (32). Helicase-dead BLM retains adaptor activity in regulating c-Myc (39). For DHX36, the ATPase-deficient E335A mutant retains nucleic acid-binding capacity (40). Despite these diverse functions, the roles, dosage sensitivity, and stress-responsive regulation of G4 helicases during aging remain poorly defined.

Using G4-affinity mass spectrometry, we identified DHX36 as enriched in aged mouse cortical lysates relative to young mice. We validated increased neuronal DHX36 expression in aged mouse and human cortex and hippocampus, with further elevation in PS19 tauopathy mice and Alzheimer’s disease (AD) brains. To identify upstream triggers, we examined whether G4 stabilization alone induces DHX36. Unexpectedly, in cultured mouse primary cortical neurons, pharmacological stabilization of G4s with pyridostatin (PDS) reduced *Dhx36* mRNA and protein levels despite increased G4-positive structures, indicating that G4 stabilization alone is not sufficient to induce DHX36 in neurons. We next tested whether aging-relevant stress regulates DHX36 expression. Pro-inflammatory cytokines (TNF-α, IL-1β, and IFN-γ), ER stress (tunicamycin and thapsigargin), and genotoxic stress (etoposide) robustly increased *Dhx36* mRNA and protein levels in cultured primary neurons and also increased G4-positive puncta. These findings indicate that DHX36 induction is associated with stress conditions rather than with G4 stabilization alone and provide an explanation for its elevation in aged and AD brains, where neurons experience chronic inflammation, ER stress, and genomic instability. To determine which transcripts are affected by increased DHX36, we overexpressed DHX36 in primary cortical neurons and performed RNA sequencing. DHX36 altered the neuronal transcriptome, suppressing inflammatory pathways and modulating genes linked to senescence, aging, and chromatin regulation. DHX36 reduced *Hmga1/2*, chromatin architectural factors that promote inflammatory and pro-senescence transcription, and HMGA1/2 protein levels were also reduced in aged mouse neurons *in vivo*. Furthermore, an ATPase-deficient DHX36 mutant (E335A) reproduced the major transcriptional repression effects of wild-type DHX36, indicating that these transcriptional effects do not require ATP-dependent unwinding. Taken together, these findings suggest that persistent neuronal stress promotes G4 accumulation and DHX36 induction, with elevated DHX36 levels contributing to transcriptional remodeling and altered inflammatory responses during aging and disease.

## Results

### The G4 helicase DHX36 is enriched in the aged mouse brain

Our previous studies showed that a putative G4-forming sequence in the *Atg7* gene (named ATG2700; d[5′AT_2_CT_2_*G*_*3*_*GCTGG*_*3*_*TC*_*3*_*T*_*2*_*GG*_*3*_*A*_*2*_*CTGTAT*_*2*_*G*_*3*_TGA_2_C_2_3′]) on the sense strand folds into a G4 *in vitro* (Gs in bold are involved in the core of the G4 structure) (41). Furthermore, we demonstrated that PC4, a transcriptional regulator and a high-affinity G4-binding protein, interacts with the ATG2700 oligonucleotide *in vitro* (41). Since the level of G4s increases in aged brains (41) and in AD (42), we hypothesized that G4s could be bound by G4-regulating proteins or helicases in the aged brain. To address this, we incubated brain extracts from the frontal cortex of both young (3 months) and aged (26 months) male mice, with a G4-DNA oligonucleotide (ATG2700) and a single-stranded (SS) DNA as a control, immobilized on beads, and analyzed them by mass spectrometry (MS). With a criterion set for >100-fold enrichment in aged brain lysates, we identified several proteins known to associate with chromatin but not previously known to bind to G4s, including ATRIP, GLYR1, and EPHA. Interestingly, we discovered that the G4 helicase DHX36 specifically bound to the ATG2700 probe. DHX36 was one of the top hits and was more enriched in aged brain lysates compared to those from young brains (Fig. 1).

**Figure 1:**
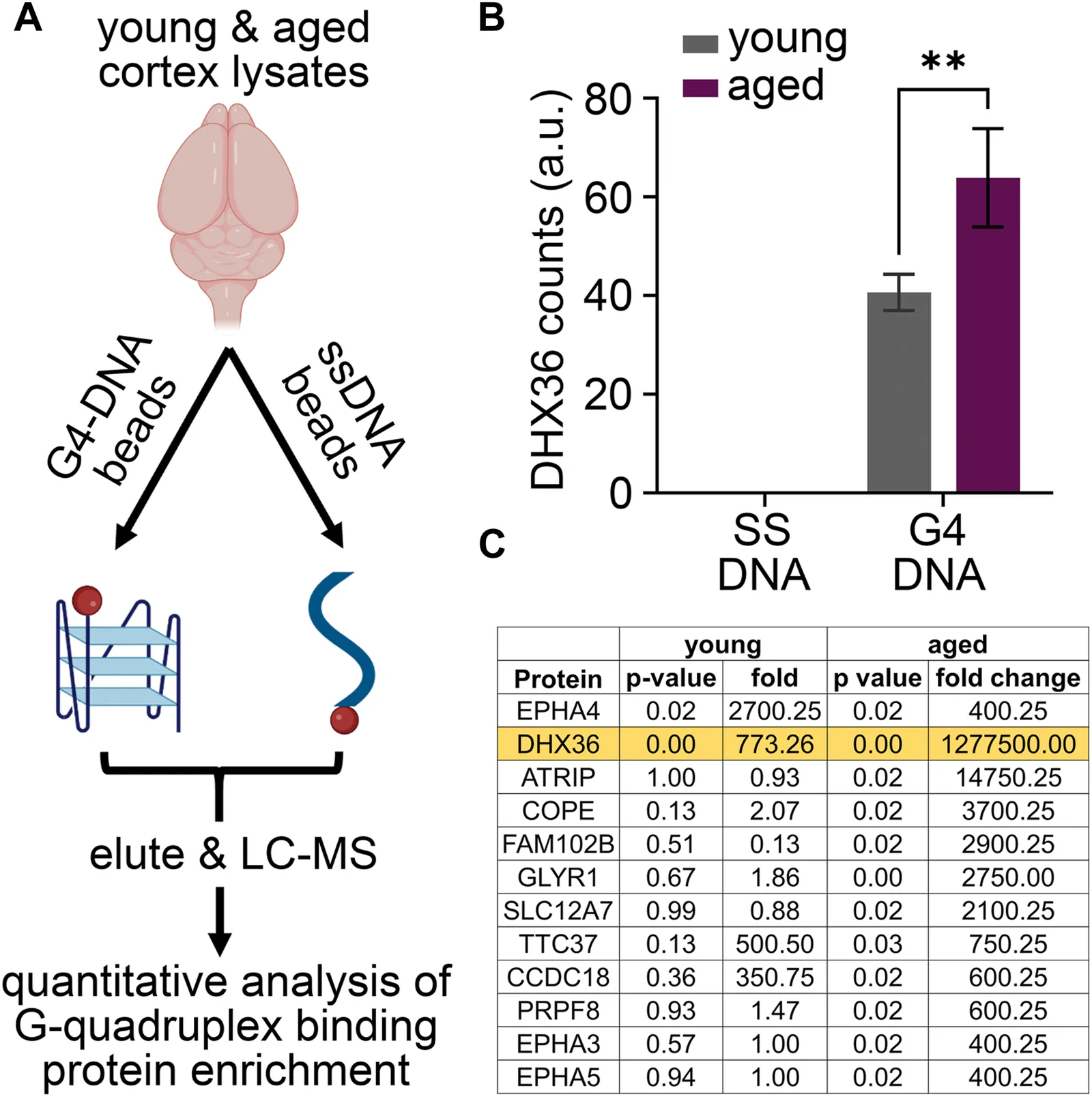
**The G4 helicase DHX36 is enriched in aged mouse brains.***A*, schematic of the pulldown-LC-MS workflow. Cortical lysates from young and aged mice were incubated with immobilized G4-DNA or single-stranded DNA (ssDNA) oligonucleotide-conjugated beads. Bound proteins were eluted and subjected to LC-MS to quantitatively identify G-quadruplex-interacting proteins. *B*, quantification of DHX36 abundance on ssDNA and G4-DNA beads from young and aged cortices. DHX36 shows significantly greater enrichment for G4-DNA oligos in aged brains than in young controls. *C*, representative list of proteins enriched on G4-DNA beads identified by LC-MS from young and aged cortices. DHX36 emerges as the top G4-binding protein in aged brains, exhibiting the largest fold change among all detected proteins. *N* = 4 biological replicates. Data represent mean ± SEM. ∗*∗p* = 0.001, two-way ANOVA.

To determine whether this reflected increased DHX36 abundance, we assessed DHX36 protein levels in young and aged wild-type brains. Mouse coronal brain sections were stained with a DHX36-specific antibody and neuronal marker NeuN. We found a significant upregulation of DHX36 expression in the neurons of aged (24 months) mice compared to young (4 months) mice in the cortex and hippocampus, in both males and females (Fig. 2). DHX36 was localized to both the nucleus and the cytoplasm, and total DHX36 intensity was measured in NeuN-positive neurons. We conclude that DHX36 expression is elevated in aged brains, establishing DHX36 as a G4-associated protein whose levels increase with aging.

**Figure 2:**
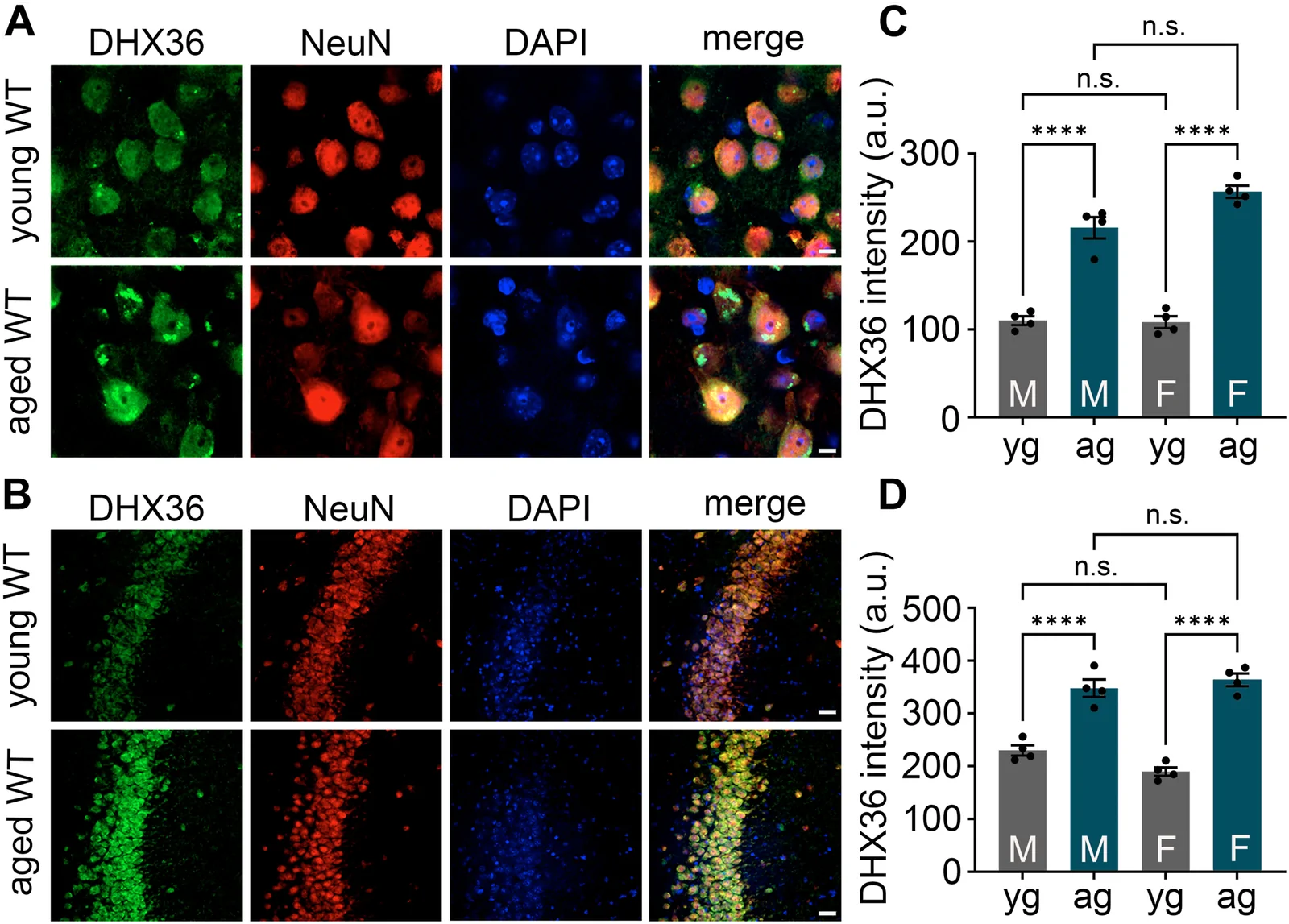
**DHX36 expression is elevated inaged mouse brains.***A*, representative confocal images of coronal cortical sections from young and aged wild-type mice stained for DHX36 (*green*), NeuN (*red*), and DAPI (*blue*). *B*, confocal images of hippocampal sections from young and aged wild-type mice showing DHX36 (*green*), NeuN (*red*), and DAPI (*blue*). *C*, quantification of DHX36 fluorescence intensity in cortical neurons demonstrates a significant age-associated increase in both male and female mice. *D*, DHX36 levels are similarly elevated in hippocampal neurons from aged mice compared with those from young controls. Fluorescence intensities were measured in ImageJ and normalized. Scale bar = 10 μm. *N* = 4 biological replicates per group (young male and female (4 months), aged male and female (24 months)). Statistical analysis was performed using one-way ANOVA. Data represent mean ± SEM. ∗∗∗∗*p* < 0.0001. n.s = not significant.

### DHX36 expression is increased in PS19 mice and in AD human brains

We asked whether DHX36 expression is altered in neurodegenerative disease contexts associated with tau pathology. To address this, we measured the levels of DHX36 in aged wild-type and aged Tau PS19 mice, a model of tauopathy relevant to AD (43, 44, 45). In male and female PS19 mice, DHX36 expression was significantly higher in the cortex and hippocampus than in age-matched (24 months) wild-type controls (Fig. 3, *A*–*D*). Interestingly, female PS19 mice exhibited higher DHX36 levels in the hippocampus than males, suggesting a sex-dependent expression pattern (Fig. 3*D*). To determine whether similar changes occur in humans, we analyzed DHX36 expression in brain tissue from the CA3 region of the hippocampus obtained from young and aged normal individuals, as well as in both male and female AD patients. In agreement with our previous observations in mice, DHX36 expression was higher in the aged *versus* young individuals (Fig. 4, *A* and *B*).

**Figure 3:**
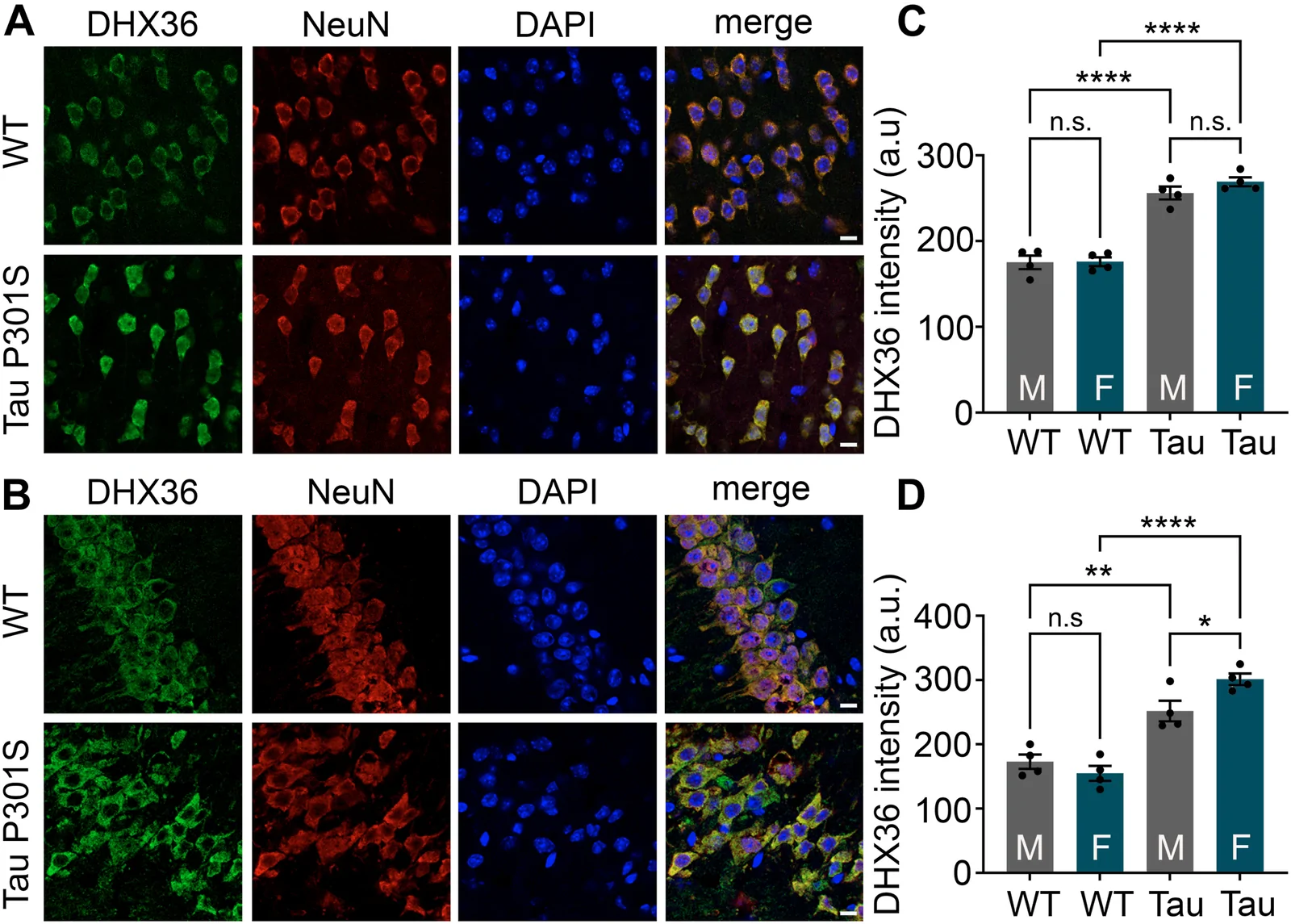
**DHX36 expression is elevated in neurons of PS19 mice.***A*, representative confocal images of cortical sections from wild-type (WT) and PS19 mice stained for DHX36 (*green*), NeuN (*red*), and DAPI (*blue*). *B*, representative hippocampal sections from WT and PS19 mice stained with DHX36, NeuN, and DAPI. *C*, quantification of DHX36 fluorescence intensity in cortical neurons shows a significant increase in PS19 mice compared to WT controls in both males and females. *D*, quantification of DHX36 levels in hippocampal neurons also demonstrates elevated DHX36 expression in PS19 mice relative to WT controls. Fluorescence measurements were performed in ImageJ and normalized. Scale bar = 10 μm. *N* = 4 biological replicates per group (male WT, female WT, male PS19, female PS19). Statistical analysis was performed using one-way ANOVA. Data represent mean ± SEM. ∗*p* < 0.05, ∗∗*p* < 0.01, ∗∗∗∗*p* < 0.0001. n.s = not significant.

**Figure 4:**
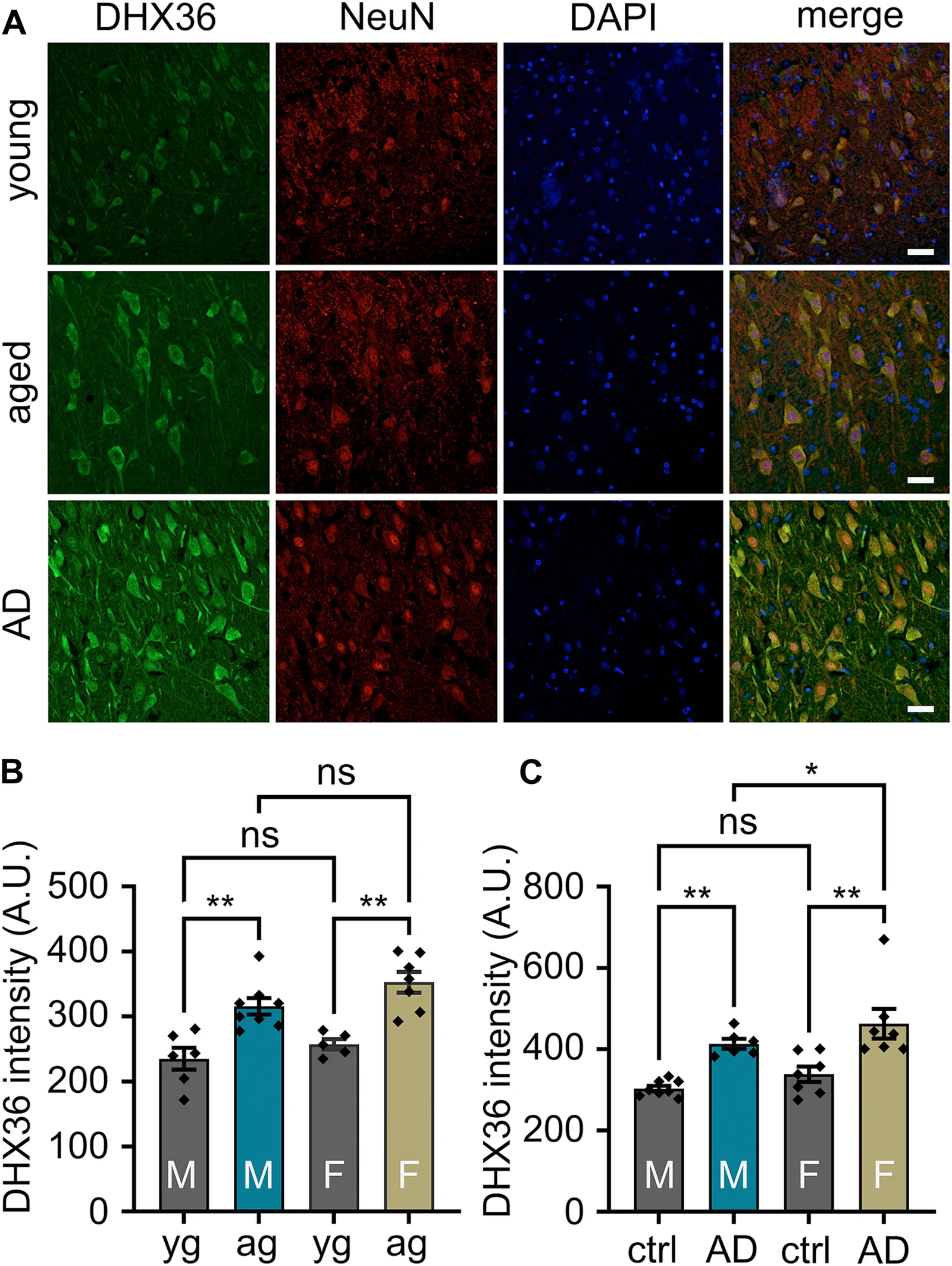
**DHX36 increases in the human hippocampal CA3 region during aging and AD.***A*, representative confocal images of human hippocampal CA3 sections from young, aged, and AD cases stained for DHX36 (*green*), NeuN (*red*), and DAPI (*blue*). The DHX36 signal appears stronger in aged and AD CA3 neurons relative to young normal individuals. *B*, quantification of DHX36 fluorescence intensity in CA3 neurons shows a significant increase in aged males and aged females compared to their young counterparts. No sex-dependent differences were observed in either the young or aged groups (ns). *C*, DHX36 intensity is significantly increased in AD males and AD females relative to sex-matched normal individuals. Fluorescence intensities were measured in ImageJ and normalized. Scale bar = 10 μm. *N* = 6 to 8 human cases per group (Table 1). Statistical analysis was performed using one-way ANOVA. Data represent mean ± SEM. ∗*p* < 0.05, ∗∗*p* < 0.01, ns = not significant.

We next assessed DHX36 expression in AD patient brains (Table 1) and found that DHX36 was further elevated in these brains (Fig. 4). DHX36 levels were higher in female than in male AD cases (Fig. 4*C*). These findings were supported at the transcript and protein levels. RT-qPCR and JESS capillary immunoassay showed that DHX36 mRNA and protein were significantly increased in aged normal individuals and AD hippocampus relative to young normal individual samples (Fig. S2). In summary, we show that DHX36 expression increases with age and is further upregulated in AD (Figs. S1 and S2). The increase in DHX36 in both aged and AD brains suggests its potential involvement in G4-mediated regulatory mechanisms that may contribute to the aging and pathology of age-related neurological disorders.

**Table 1:** Demographic and neuropathological characteristics of human brain donors

Group	Sex	Age	Race	Neuropathology report
Young control individuals	Male N = 6	50	AA	no neurodegenerative pathologies
		39	Hispanic	no neurodegenerative pathologies
		21	Hispanic	no neurodegenerative pathologies
		23	Caucasian	no neurodegenerative pathologies
		40	Caucasian	no neurodegenerative pathologies
		42	AA	no neurodegenerative pathologies
	Female N = 6	16	Hispanic	no neurodegenerative pathologies
		54	Hispanic	no neurodegenerative pathologies
		38	AA	no neurodegenerative pathologies
		33	AA	no neurodegenerative pathologies
		35	AA	no neurodegenerative pathologies
		32	Hispanic	no neurodegenerative pathologies
AD individuals	Male N = 6	82	Caucasian	A3B3C3 + Tauopathy + CAA
		84	Caucasian	A3B3C3
		85	Caucasian	A3B3C2 + LBD + CAA
		83	AA	A3B3C3 + CAA
		95	Caucasian	A3B3C3 + LATE + NC
		79	AA	A3B3C3 + CAA
	Female N = 7	93	Caucasian	A3B2C3
		81	Caucasian	A3B2C3
		82	AA	A3B3C3 + CAA
		86	AA	A3B3C3 + CAA
		64	Caucasian	A3B2C3
		73	Hispanic	A3B2C3
		86	Hispanic	A3B2C3 + CAA
aged control individuals	Male N = 8	82	Asian	no neurodegenerative pathologies
		75	AA	no neurodegenerative pathologies
		72	AA	no neurodegenerative pathologies
		62	Hispanic	no neurodegenerative pathologies
		64	AA	no neurodegenerative pathologies
		61	Hispanic	PART
		67	AA	PART
		61	Caucasian	no neurodegenerative pathologies
	Female N = 7	74	Asian	age-related changes
		80	Hispanic	age-related changes
		73	AA	PART
		68	Caucasian	no neurodegenerative pathologies
		71	AA	age-related changes
		77	Caucasian	age-related changes
		55	AA	PART

*Postmortem* hippocampal tissue was obtained from young control individuals (*n* = 12; six male, six female; ages 16–54 years), aged control individuals (*n* = 15; eight male, seven female; ages 55–82 years), and individuals with Alzheimer's disease (*n* = 13; six male, seven female; ages 64–95 years). Sex, age at death, race, and the neuropathology report are listed for each case. Neuropathological diagnoses for Alzheimer's disease, including the ABC score comprising the amyloid-β plaque score (A), neurofibrillary tangle score (B), and neuritic plaque score (C), as well as cerebral amyloid angiopathy (CAA) and Lewy body disease (LBD), were determined according to the National Institute on Aging–Alzheimer's Association guidelines for the neuropathologic assessment of Alzheimer's disease (81). Limbic-predominant age-related TDP-43 encephalopathy neuropathologic changes (LATE-NC) were assessed based on the 2019 international consensus report (82), and primary age-related tauopathy (PART) was diagnosed according to the 2014 consensus criteria (83). AA, African American.

### Stabilization of G4s does not upregulate DHX36 expression in neurons

Our recent study showed that the stabilization of G4s by PDS alters neuronal transcription, influencing pathways involved in p53 signaling, immune responses, and cellular senescence (46). Unexpectedly, *Dhx36* was not upregulated in our PDS-treated neurons (46). This was surprising because PDS increased G4-positive structures in neurons, and since G4s are the substrate of DHX36, we expected an increased G4 load to elicit a compensatory increase in DHX36 expression. The absence of *Dhx36* induction in our previously published neuronal RNA-seq dataset, therefore, prompted us to validate this result independently in the present study. We assessed DHX36 expression and found that PDS treatment did not increase DHX36 expression but instead reduced it in neurons compared to controls (Fig. 5, *A* and *B*). We next quantified *Dhx36* mRNA by RT-qPCR and DHX36 protein by JESS capillary immunoassay, revealing that both mRNA and protein were significantly downregulated in PDS-treated neurons compared to vehicle-treated controls (Fig. 5, *C*–*E*). These data confirmed that the lack of *Dhx36* induction after PDS treatment was reproducible at both the transcript and protein levels. These results indicate that G4 stabilization alone does not upregulate DHX36 expression in neurons, despite increased G4 load, in contrast to the elevated DHX36 levels observed in aged and AD brains. PDS differs from these stressors in acting directly on nucleic acids rather than through signaling pathways, which led us to investigate whether DHX36 induction occurs under stress conditions that engage signaling pathways relevant to aging.

**Figure 5:**
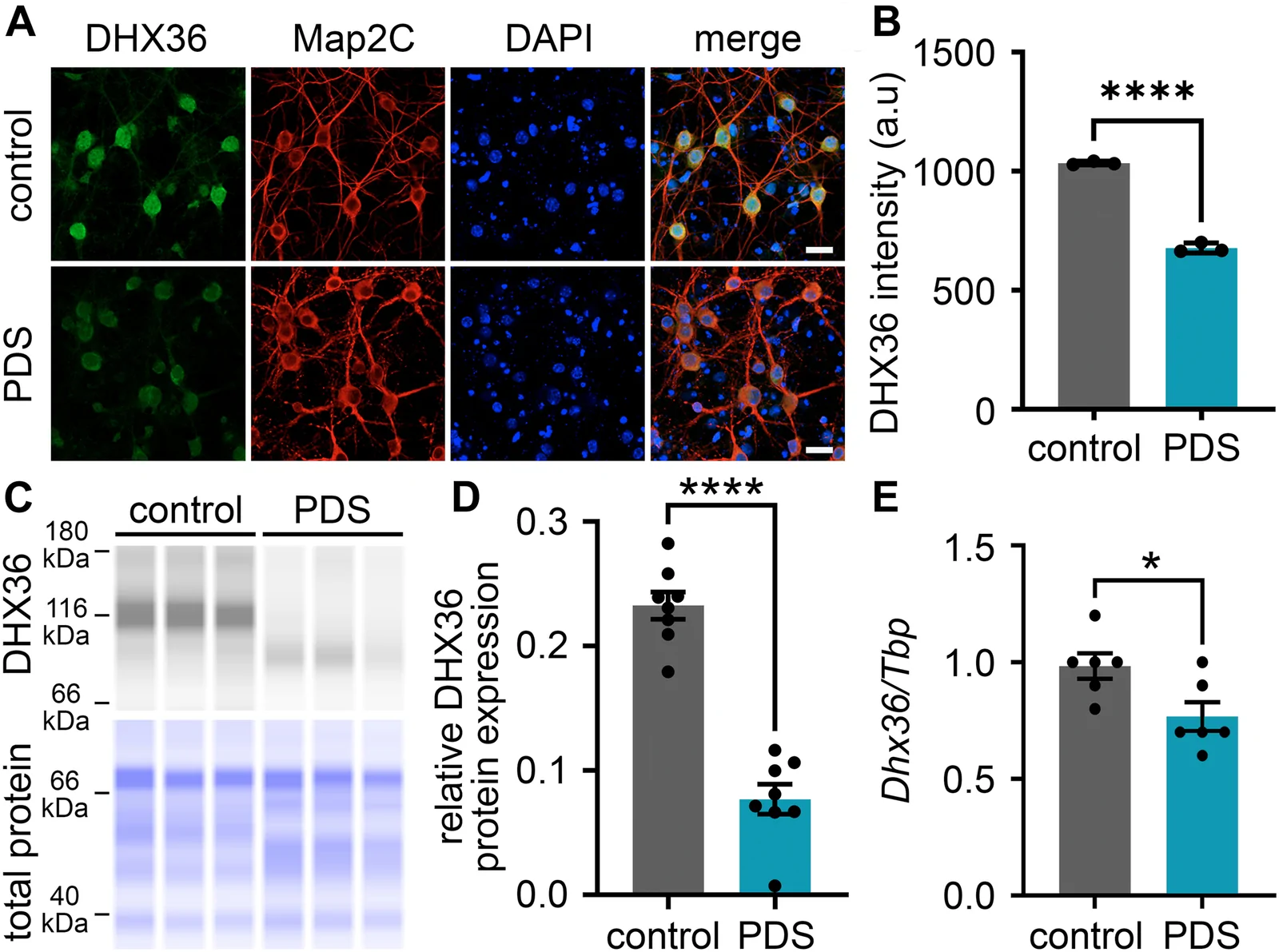
**G4-stabilization by PDS reduces DHX36 expression in neurons.***A*, representative confocal images of primary mouse neurons subjected to G4 stabilization stress using PDS (2 μM). Neurons were stained for DHX36 (*green*), MAP2C (*red*), and DAPI (*blue*). PDS treatment significantly reduced DHX36 intensity compared with vehicle-treated controls. *B*, quantification of DHX36 fluorescence intensity confirms a strong reduction in DHX36 levels following PDS treatment, N = 3. *C*, representative JESS capillary immunoassay output for DHX36 from control and PDS-treated neurons, with total-protein normalization. *D*, quantification of the JESS signal shows a significant reduction in DHX36 protein expression after PDS treatment, N = 8. *E*, qPCR analysis of *Dhx36* (normalized to Tbp) shows that G4-induced stress also reduces *Dhx36* mRNA levels. Primary cortical neurons were treated at DIV14, N = 6. Data represent mean ± SEM. Statistical analysis was performed using an unpaired two-tailed *t*-test. ∗*p* < 0.05, ∗∗∗∗*p* < 0.0001. Scale bar = 10 μm.

### Stress-associated signaling upregulates DHX36 expression and increases G4-positive puncta in neurons

To identify upstream signals that may contribute to the elevated levels observed in aged and AD brains, we exposed neurons to a range of stress conditions relevant to aging. Neurons were treated with pro-inflammatory cytokines TNF-α, IL-1β, and IFN-γ, ER stress inducers tunicamycin and thapsigargin, and the DNA-damaging agent etoposide. These stressors model persistent inflammatory, proteostatic, and genotoxic challenges characteristic of the aging brain. In all six conditions, we observed a robust increase in DHX36 expression (Fig. 6). Immunocytochemistry revealed an elevated DHX36 signal compared to untreated controls, with increased nuclear accumulation in all stress conditions (Fig. 6, *A*–*C*). RT-qPCR confirmed that *Dhx36* mRNA levels were significantly higher, and JESS capillary immunoassay demonstrated a corresponding increase in DHX36 protein in all stress paradigms (Fig. 6, *D*–*H*). These findings demonstrate that DHX36 is induced by inflammatory and ER stressors, as well as DNA damage signals, in neurons, which are prominent features of aging and neurodegeneration. Together with the PDS experiment, these findings indicate that DHX36 induction is associated with signaling-active stress conditions rather than with G4 stabilization alone.

**Figure 6:**
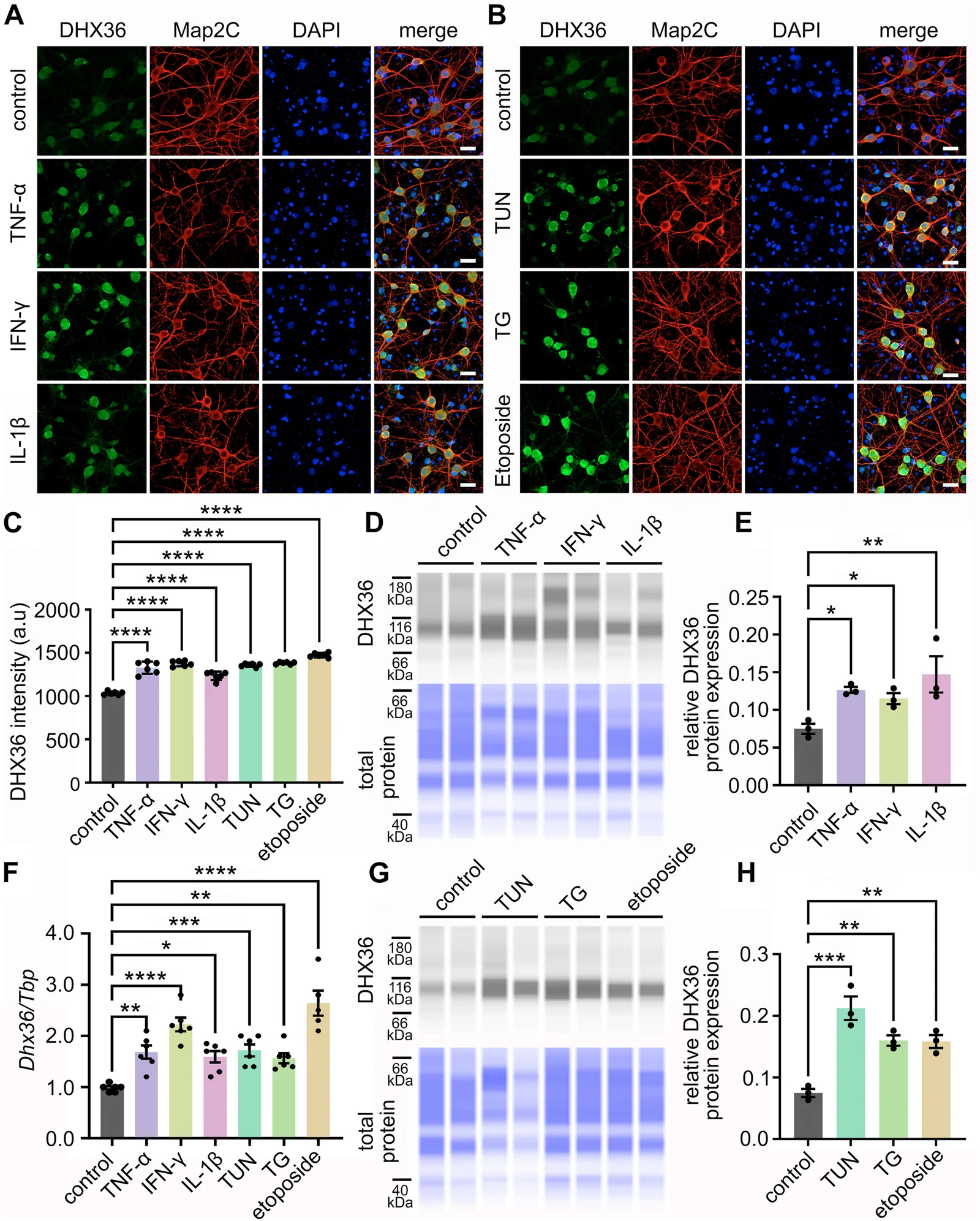
**Cellular stressors increase DHX36 expression in neurons.***A*, representative confocal images of primary mouse neurons exposed to inflammatory stress for 24 h using TNF-α (10 ng/ml), IFN-γ (10 ng/ml), or IL-1β (10 ng/ml), followed by staining for DHX36 (green), MAP2C (red), and DAPI (blue). All three pro-inflammatory cytokines significantly increased DHX36 intensity. *B*, representative confocal images of neurons treated with stressors, tunicamycin (TUN, 1 μM), thapsigargin (TG, 1 μM), or etoposide (5 μM). Each of these stressors increased DHX36 levels relative to control. All treatment conditions shown in panels *A* and *B* were performed within the same experiment and shared a single untreated control group. The same representative control images are therefore shown in both panels, allowing each set of treatment images to be compared directly with its control. *C*, quantification of DHX36 fluorescence intensity under inflammatory and ER/DNA damage stress conditions reveals a significant elevation in DHX36 levels in response to all stress stimuli (N = 6). *D*, representative JESS capillary immunoassay output for DHX36 following inflammatory stress (TNF-α, IFN-γ, IL-1β), normalized to total protein. *E*, quantification of the JESS signal reveals a significant increase in DHX36 protein expression under inflammatory stress (N = 3). *F*, qPCR analysis of *Dhx36* mRNA (normalized to Tbp) shows significant transcriptional upregulation in response to inflammatory stimuli (N = 6). *G*, representative JESS assay output for DHX36 after ER stress (TUN, TG) and DNA-damage stress (etoposide), normalized to total protein. *H*, quantification reveals increased DHX36 protein expression following ER stress or DNA damage, N = 3. All treatments were applied to DIV14 primary mouse neurons for 24 h to model cellular stress. Data represent mean ± SEM. ∗*p* < 0.05, ∗∗*p* < 0.01, ∗∗∗*p* < 0.001, ∗∗∗∗*p* < 0.0001, one-way ANOVA with multiple comparisons. Scale bar = 10 μm.

The effect of the same stress conditions (TNF-α, IL-1β, IFN-γ, tunicamycin, thapsigargin, and etoposide) on the formation of G4s was evaluated using N-TASQ (2, 47) and BG4 staining (6). Both probes showed the same stress-associated increase in nuclear G4 puncta (Figs. 7 and S8). The use of these two structurally unrelated probes is supported by the direct side-by-side comparison of BG4 and TASQ-based probes, which showed their complementary G4-recognition properties (47). All conditions produced a significant increase in nuclear G4 puncta relative to control neurons (Fig. 7), indicating that inflammation, ER stress, and DNA damage promote G4 formation in neurons. As a control, neurons treated with PDS also showed an increase in G4 puncta, consistent with G4 stabilization (Fig. 7, *C* and *D*). The parallel increase in G4 structures and DHX36 expression in all signaling-driven stress treatments supports a model in which inflammatory, proteostatic, and genotoxic stress may promote G4 stabilization in neurons, coinciding with elevated DHX36 expression under these conditions. These data indicate that stress-associated signaling is accompanied by increased G4-positive structures along with elevated DHX36 expression.

**Figure 7:**
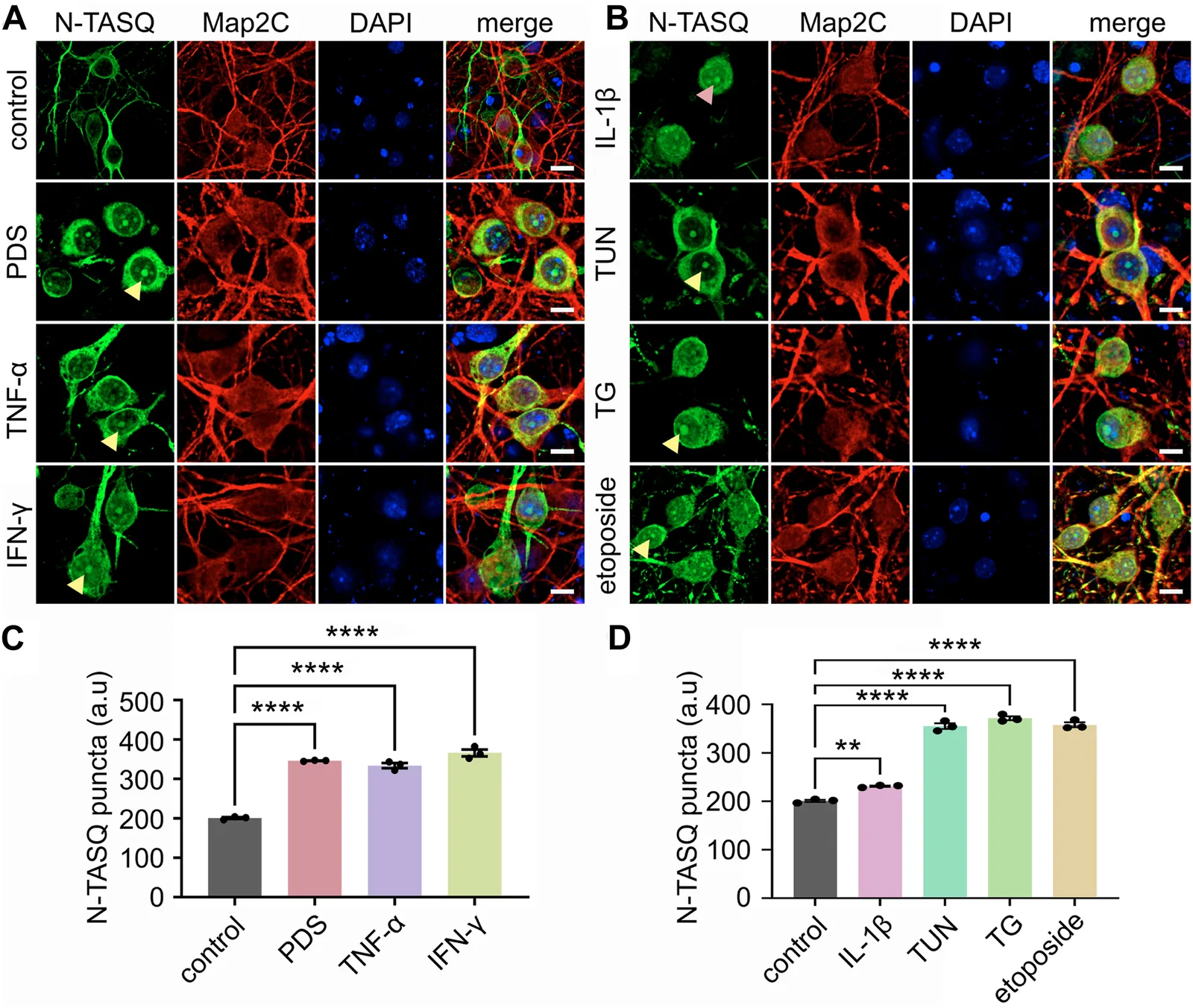
**Cellular stressors increase N-TASQ-positive puncta in neurons.***A*, representative confocal images of primary mouse neurons treated with G4 stabilization (PDS, 2 μM) or inflammatory stressors (TNF-α, 10 ng/ml; IFN-γ, 10 ng/ml) and stained with the G-quadruplex probe N-TASQ (*green*), MAP2C (*red*), and DAPI (*blue*). All stress conditions increased N-TASQ fluorescence and puncta formation (yellow arrowheads), indicating elevated neuronal G4 accumulation compared to controls. *B*, representative images of neurons treated with IL-1β (10 ng/ml), ER stressors tunicamycin (TUN, 1 μM) or thapsigargin (TG, 1 μM), or DNA-damage stress (etoposide, 5 μM). Each treatment enhanced the N-TASQ puncta signal, consistent with the increased formation of G4 structures. *C*, quantification of N-TASQ puncta intensity under PDS and inflammatory stress (TNF-α, IFN-γ) shows a robust increase in neuronal G4 formation. *D*, quantification of N-TASQ puncta intensity following IL-1β, ER stressors (TUN, TG), and etoposide shows a significant elevation in G4 levels. All treatments were applied for 24 h at DIV14 to model PDS or cellular stress. Data represent mean ± SEM. N = 3. ∗∗*p* < 0.01, ∗∗∗∗*p* < 0.0001, one-way ANOVA with multiple comparisons. Scale bar = 10 μm.

### DHX36 regulates transcription in neurons

We found that DHX36 expression was elevated in the cortex and hippocampus of aged brains compared to their younger counterparts (Fig. 2). Given that DHX36 regulates transcription (48, 49, 50), and that DHX36 levels were elevated in aged and AD brains and under stress conditions, we next sought to determine which transcriptional programs are influenced by increased DHX36 expression in neurons. To address this, we cultured neurons and infected them with lentiviruses carrying either the control mScarlet, mScarlet-tagged DHX36, or mScarlet-mutant DHX36 (E335A), and analyzed all three groups simultaneously. The mScarlet and mScarlet-DHX36 signals colocalized with the neuronal marker MAP2C (Fig. S7, *A* and *B*), and RT-qPCR together with JESS capillary immunoassay confirmed increased *Dhx36* transcript and DHX36 protein levels in neurons infected with mScarlet-DHX36 (∼6-fold and 2-fold, respectively; Fig. S7, *F* and *I*). Cells were subjected to RNA-sequencing analysis, and the Principal Component Analysis (PCA) revealed distinct patterns between the control samples and those infected with the mScarlet-DHX36 lentivirus (Fig. 8*A*): 286 differentially expressed genes (DEGs) were identified out of a pool of 18,053 genes with measurable expression levels, with 69 DEGs upregulated and 217 downregulated (Fig. 8, *B*–*E*). The most strongly upregulated genes included *Il23r*, *Pagr1a*, *Npb*, *Defb11*, and *Drd3*, along with Dhx36 itself (log2 fold change 2.1, ∼4.3-fold), confirming overexpression (Fig. 8*D*). The most strongly downregulated genes included *Ccl2*, *Lcn2*, *Mmp3*, *Bcl2a1a*, and *Dnm3os* (Fig. 8*E*). These results show that DHX36 induces substantial differential gene expression in neurons, impacting both positively and negatively regulated genes.

**Figure 8:**
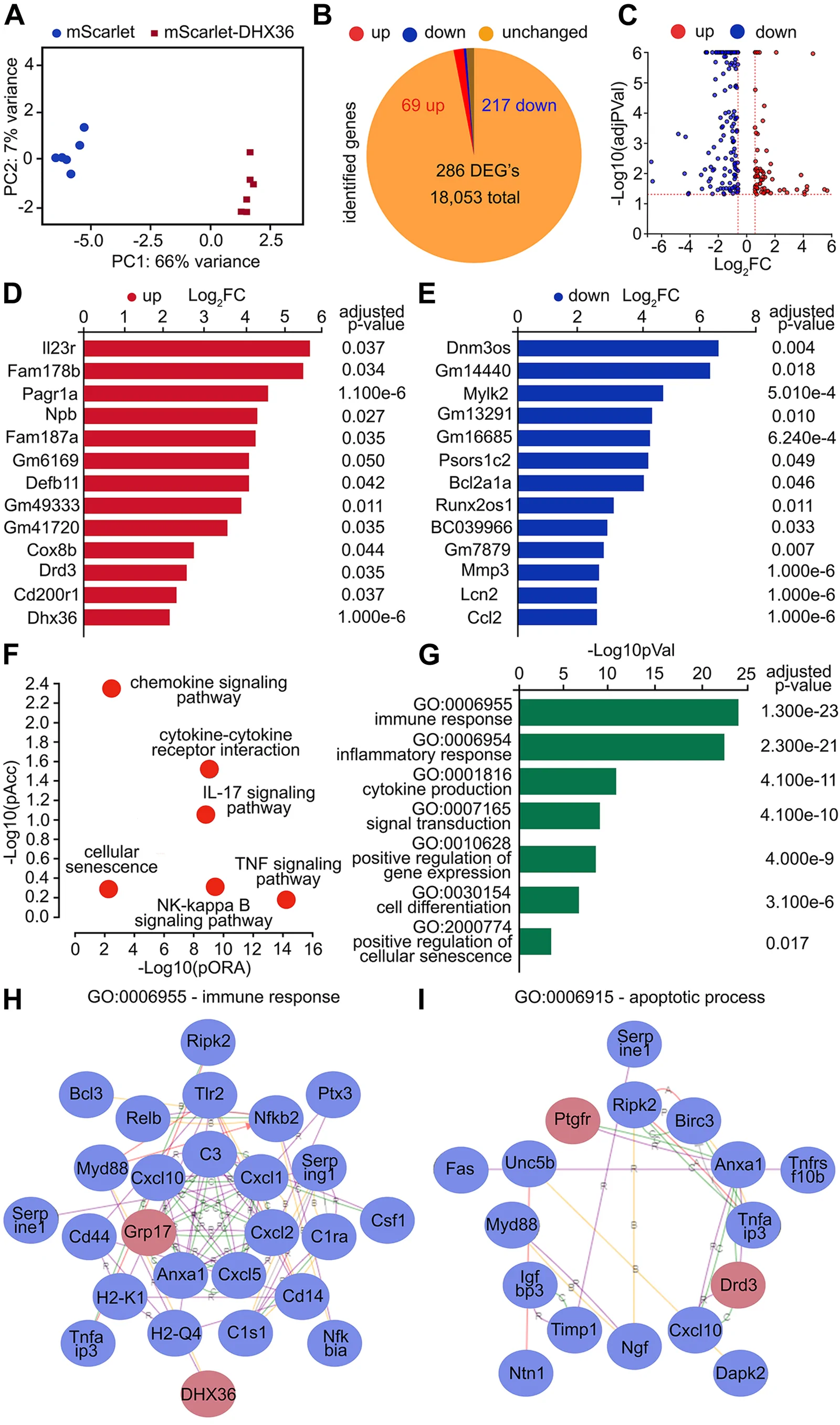
**DHX36 regulates transcriptional programs linked to immune activation, cytokine signaling, and cellular senescence in neurons.***A*, principal component analysis (PCA) of RNA seq profiles from neurons expressing mScarlet or mScarlet-DHX36. Each point represents an individual sample, and the percentages indicate the variance explained by each principal component. DHX36 overexpression induces a clear transcriptional shift. *B*, Pie chart summarizing the 286 differentially expressed genes (DEGs) identified from 18,053 genes, with 69 genes upregulated and 217 downregulated upon DHX36 overexpression |log_2_FC| ≥ 0.6 with FDR < 0.05 throughout. *C*, Volcano plot illustrating all DEGs, upregulated genes (*red*), and downregulated genes (*blue*). Dashed lines indicate the fold-change and significance thresholds used for DEG selection. *D*, top upregulated DEGs ranked by log_2_ fold change, highlighting genes involved in cytokine production, innate immune responses, and stress signaling. *E*, top downregulated DEGs ranked by log_2_ fold change, including genes associated with apoptosis, chromatin regulation, and neuronal homeostasis. *F*, pathway perturbation *versus* over-representation analysis (pAcc *versus* pORA) identifies key pathways altered by DHX36, including chemokine signaling, cytokine–cytokine receptor interaction, IL-17 signaling, NF-κB signaling, TNF signaling, and cellular senescence. *G*, Gene Ontology (GO) over-representation analysis of DEGs reveals significant enrichment in immune response, inflammatory response, cytokine production, signal transduction, cell differentiation, and positive regulation of cellular senescence. *H*, network visualization of DEGs associated with the GO term immune response (GO:0006955). DHX36 overexpression alters a coordinated module of immune-regulatory genes, including Ccl2, Cxcl2, Myd88, Il1r1, and Ripk families. *I*, network analysis of DEGs enriched in the GO term apoptotic process (GO:0006915), showing DHX36-dependent regulation of pro- and anti-apoptotic genes such as Fas, Bcl2l1, Drd3, Ntn1, and Timp1.

The RNA-sequencing data were then examined in the framework of pathways sourced from the Kyoto Encyclopedia of Genes and Genomes (KEGG) database and gene ontologies from the Gene Ontology (GO) Consortium database. We extracted pathway topologies, encompassing genes and their directional interactions, and found that the prominent enriched pathways included cytokine-cytokine receptor interaction, IL-17 signaling, NF-κB signaling, TNF signaling, PI3K-AKT signaling, MAPK signaling, p53 signaling, cellular senescence, signal transduction, regulation of gene expression, and the apoptotic process, among others (Fig. 8, *F* and *G*). To summarize, our RNA-sequencing data highlight the significant impact of DHX36 on transcriptional changes associated with genes involved in immune, stress, and inflammatory responses in neuronal cells. Notably, the majority of these transcriptional changes reflected gene suppression, consistent with DHX36 acting as a negative regulator of inflammatory and stress-associated transcriptional programs.

### DHX36 alters immune response genes

It was reported that DHX36 plays a crucial role in regulating processes related to immune responses in non-neuronal cells (51, 52, 53, 54). Consistent with this, our RNA-seq analysis identified DHX36 as a modulator of genes associated with the immune response (GO:0006955) and chemokine signaling pathway (KEGG:04062), exerting differential regulatory effects on 73 genes. The dominant transcriptional effect of DHX36 is gene suppression, with 66 immune-related genes downregulated and only seven upregulated, and some examples include *Il23r* (cytokine receptor), *Pagr1a* (chromatin factor)*, Defb11* (antimicrobial peptide)*, Gpr17* (damage-response receptor)*, Npb* (neuropeptide B), and *Ulbp1* (NK-ligand/immune activator). All the genes associated with the CXCL family, including *Cxcl1, Cxcl2, Cxcl5, and Cxcl10,* were substantially downregulated by DHX36 (Fig. 8*H*). Other downregulated genes included *Bcl3* (NF-κB regulator), *Tlr2* (pattern-recognition receptor)*, Foxf1* (transcription factor)*, Gbp2* (interferon effector)*, Cd14* (innate immune receptor), *Cd44* (cell-adhesion receptor)*, Myd88* (immune adaptor), *Ddx60* (antiviral helicase), and *Parp14* (immune regulator) (Fig. 8*H*).

To validate these transcriptomic findings, we measured ten differentially expressed genes by RT-qPCR in neurons expressing mScarlet or mScarlet-DHX36. Consistent with the RNA-seq data, *Cxcl1*, *Casp4*, *Il19*, *Tlr2*, *Bcl3*, *Serpine1*, *Cd14*, and *Myd88* were significantly reduced, and *Pagr1a* and *Gpr17* were increased, in DHX36-overexpressing neurons (Fig. S9). In the realm of immune signaling, DHX36 negatively regulates MyD88, a crucial adaptor protein in inflammatory pathways downstream of Toll-like receptors (TLRs) and the interleukin-1 (IL-1) receptor families (53). These differentially expressed genes largely fall into chemokine signaling, Toll-like receptor signaling, and interferon-associated immune pathways.

### DHX36 affects genes related to apoptosis, ferroptosis, and senescence

DHX36 plays a regulatory role in genes associated with the apoptotic process (GO:0006915) and ferroptosis (KEGG:04216). These pathways are closely linked to neuronal vulnerability and stress-induced cell death during aging and neurodegeneration. Within this set, seven genes were upregulated and 44 downregulated (Figs. 8*I* and S3). *Bmf*, a pro-apoptotic Bcl-2 modifying factor implicated in various pathological conditions such as AD, seizures, cancer, and autoimmunity, is upregulated by DHX36 (55). Among the downregulated genes are *Bcl2a1a, Casp4, Il19, Bcl3, Birc3, Sphk1, Unc5b,* and others. The downregulation of Caspase-4, implicated in ER-stress-induced apoptosis and β-amyloid-induced cell death, is particularly noteworthy (56). The genes associated with ferroptosis, such as Cp (ceruloplasmin), Slc39a14, and Slc7a11, were downregulated.

During aging, cells undergo extensive chromatin-associated epigenetic modifications that influence their physiological state and drive differential gene expression (57, 58, 59, 60). Our RNA-seq analysis also indicated that DHX36 downregulated key genes associated with cellular senescence and the positive regulation of aging, including *Gadd45b, Serpine1, Cdkn2b,* and *Igfbp3* (Fig. 8). To validate these observations, we measured representative senescence-linked targets by RT-qPCR in neurons expressing mScarlet or mScarlet-DHX36. Consistent with the RNA-seq analysis, *Serpine1* was reduced in DHX36-overexpressing neurons (Fig. S9). In summary, these findings suggest that DHX36 modulates age-associated and stress-responsive pathways, with a predominant effect in suppressing pro-apoptotic and pro-senescence gene programs.

### DHX36 alters the expression of high-mobility group proteins HMGA1 and HMGA2

Our RNA-seq analysis revealed that DHX36 downregulated the expression of *Hmga1* and *Hmga2*, which encode HMGA1 and HMGA2, non-histone chromatin-binding proteins that modulate chromatin architecture and gene regulation (61, 62). Gene ontology terms associated with *Hmga1* and *Hmga2* include aging, cellular senescence, chromatin organization, and transcription regulation (Fig. S4). HMGA proteins maintain an open, transcriptionally permissive chromatin state by binding to AT-rich regions of DNA and influencing the accessibility of transcription factors, promoting the expression of inflammatory and senescence-associated genes (62, 63, 64, 65, 66). To validate our findings, we infected neurons with lentiviruses encoding mScarlet or mScarlet-DHX36. Consistent with the transcriptomic data, ectopic expression of DHX36 reduced both RNA and protein levels of HMGA1 and HMGA2 (Fig. S7). Immunocytochemistry further confirmed that HMGA proteins localized to the nucleus, and overexpression of DHX36 reduced nuclear HMGA1 and HMGA2 levels in neurons (Fig. S7, *A* and *B*). To investigate whether this regulatory relationship is also reflected in aging, we assessed HMGA1 and HMGA2 protein expression in the brains of young and aged wild-type mice. We used immunohistochemical analysis to measure HMGA protein levels, and mouse coronal brain sections were stained with HMGA1 and HMGA2-specific antibodies. We observed a significant downregulation of HMGA1 (Fig. S5) and HMGA2 (Fig. S6) expression in the brains of aged (24 months) mice compared to those of young (4-month-old), in both the cortex and hippocampus, in both males and females. Notably, in the hippocampus, aged female brains exhibit reduced HMGA1 protein levels compared to those of males. These findings show that DHX36 overexpression reduces HMGA1 and HMGA2 levels in neurons.

### Repression of DHX36 target genes does not require ATPase activity

To test whether the transcriptional effects of DHX36 require its catalytic activity, we generated a lentivirus expressing mScarlet-tagged human DHX36 carrying the E335A substitution. This residue lies within the DEAH box (residues 334–337) in the RecA1 domain, and its mutation abolishes ATP hydrolysis, while the DHX36-specific motif and the region required for G4-DNA and G4-RNA binding (residues 53–105) remain intact (40, 67, 68) (Fig. 9*A*). Our RNA sequencing experiment included three groups processed in parallel: primary cortical neurons expressing mScarlet, wild-type mScarlet-DHX36, or mScarlet-DHX36-E335A. The mScarlet lentivirus served as the control for both DHX36 constructs.

**Figure 9:**
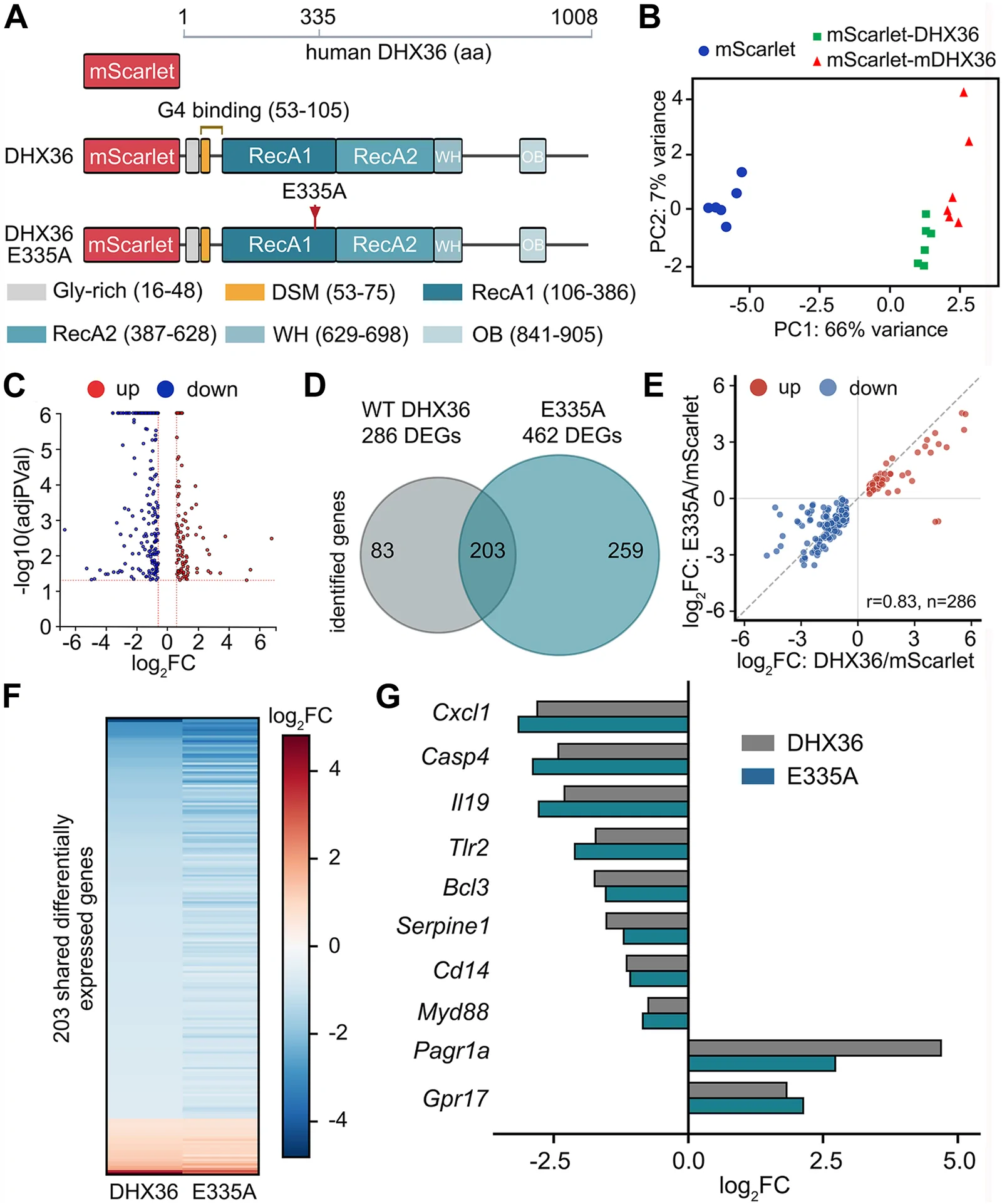
**An ATPase-deficient DHX36 mutant reproduces the major transcriptional effects of wild-type DHX36.***A*, Lentiviral constructs used in this study. mScarlet was fused to the N-terminus of full-length human DHX36 (isoform 1, NM_020865.3). The E335A substitution lies within the DEAH box (residues 334–337) in RecA1 and abolishes ATP hydrolysis, whereas the DHX36-specific motif (DSM) and the region required for G4-DNA and G4-RNA binding (residues 53–105) are unaffected. Domain boundaries are from UniProt Q9H2U1. *B*, principal component analysis of RNA sequencing data from primary cortical neurons infected with mScarlet, mScarlet-DHX36, or mScarlet-DHX36-E335A, showing separation of the three groups along PC1. *C*, Volcano plot of differential gene expression in mScarlet-DHX36-E335A relative to mScarlet. *Red*, upregulated; *blue*, downregulated. Dashed lines indicate the significance thresholds. *D*, overlap between the genes regulated by wild-type DHX36 and by E335A relative to mScarlet, among the 18,053 genes measured. Of the 286 genes regulated by wild-type DHX36, 203 were also regulated by E335A, a 28-fold enrichment over the 7.3 expected by chance (hypergeometric test, *p* < 1 × 10^−100^). *E*, Log_2_ fold change relative to mScarlet for the 286 genes regulated by wild-type DHX36, plotted for wild-type against E335A. *Red*, upregulated by wild-type DHX36; *blue*, downregulated; *dashed line*, identity. Pearson *r* = 0.83, *p* = 3.4 × 10^−74^. *F*, Log_2_ fold change for the 203 genes differentially expressed with both constructs, ranked by the wild-type value; all 203 changed in the same direction with both constructs. *G*, RNA sequencing log_2_ fold changes for the ten genes selected for RT-qPCR validation. Differentially expressed genes were defined as |log_2_FC| ≥ 0.6 with FDR < 0.05 throughout. *N* = 6 independent cultures per group.

Principal component analysis separated E335A-expressing neurons from mScarlet controls along PC1 (Fig. 9*B*), and differential expression analysis identified 462 genes altered by E335A relative to mScarlet (117 up, 345 down; |log_2_FC| ≥ 0.6, FDR < 0.05) (Fig. 9*C*), compared with 286 genes for wild-type DHX36 (69 up, 217 down) (Fig. 8). E335A reproduced the major transcriptional effects of wild-type DHX36. Of the 286 genes regulated by wild-type DHX36, 203 (71.0%) were also differentially expressed with E335A, a 28-fold enrichment over the 7.3 expected by chance (hypergeometric test, *p* < 1 × 10^−100^) (Fig. 9*D*). Fold changes produced by the two constructs were closely correlated across all 286 wild-type target genes (Pearson *r* = 0.83, *p* = 3.4 × 10^−74^) (Fig. 9*E*), and 284 of 286 (99.3%) changed in the same direction, irrespective of significance in the E335A dataset. All 203 shared genes changed in the same direction with both constructs (Fig. 9*F*).

The inflammatory and senescence-associated targets identified above behaved similarly with both constructs, including *Cxcl1*, *Casp4*, *Il19*, *Tlr2*, *Bcl3*, *Serpine1*, *Cd14*, *Myd88*, *Pagr1a*, and *Gpr17* (Fig. 9*G*), and RT-qPCR for these ten genes showed the same pattern, with wild-type DHX36 and E335A producing changes in the same direction for every gene (Fig. S9). These data indicate that the major transcriptional effects of DHX36 in neurons do not require ATP-dependent unwinding. Several helicases carry out functions that do not require ATPase activity, and the ATPase-deficient E335A mutant retains nucleic acid-binding capacity despite being unable to hydrolyze ATP (40, 67, 69).

## Discussion

A key finding of this study is that DHX36 expression in neurons is differentially regulated by small-molecule G4 stabilization *versus* aging-relevant cellular stress. Chronic stress conditions associated with aging, including inflammatory, proteostatic, and genotoxic challenges, increased *Dhx36* transcript and protein levels in neurons. In contrast, small-molecule stabilization of G4s by PDS increased nuclear G4 *foci* but did not elevate DHX36, instead reducing its expression. These findings indicate that DHX36 is not directly induced by G4 stabilization alone but is instead induced under aging-relevant stress conditions. As aging neurons experience persistent inflammation, ER and genotoxic stress, increased DHX36 expression may reflect an adaptive response to limit excessive inflammatory signaling. Aging-relevant stress combines increased G4 levels with inflammatory, proteostatic, and genotoxic signaling that promotes upregulation of DHX36 (Fig. 10). We propose that G4 accumulation occurs in parallel with cellular stress mechanisms that induce DHX36 expression, consistent with a coordinated response to stress-associated nucleic acid dysregulation rather than a direct G4-driven induction mechanism. DHX36 thus appears to be a stress-inducible helicase modulated under stress conditions in aging neurons.

**Figure 10:**
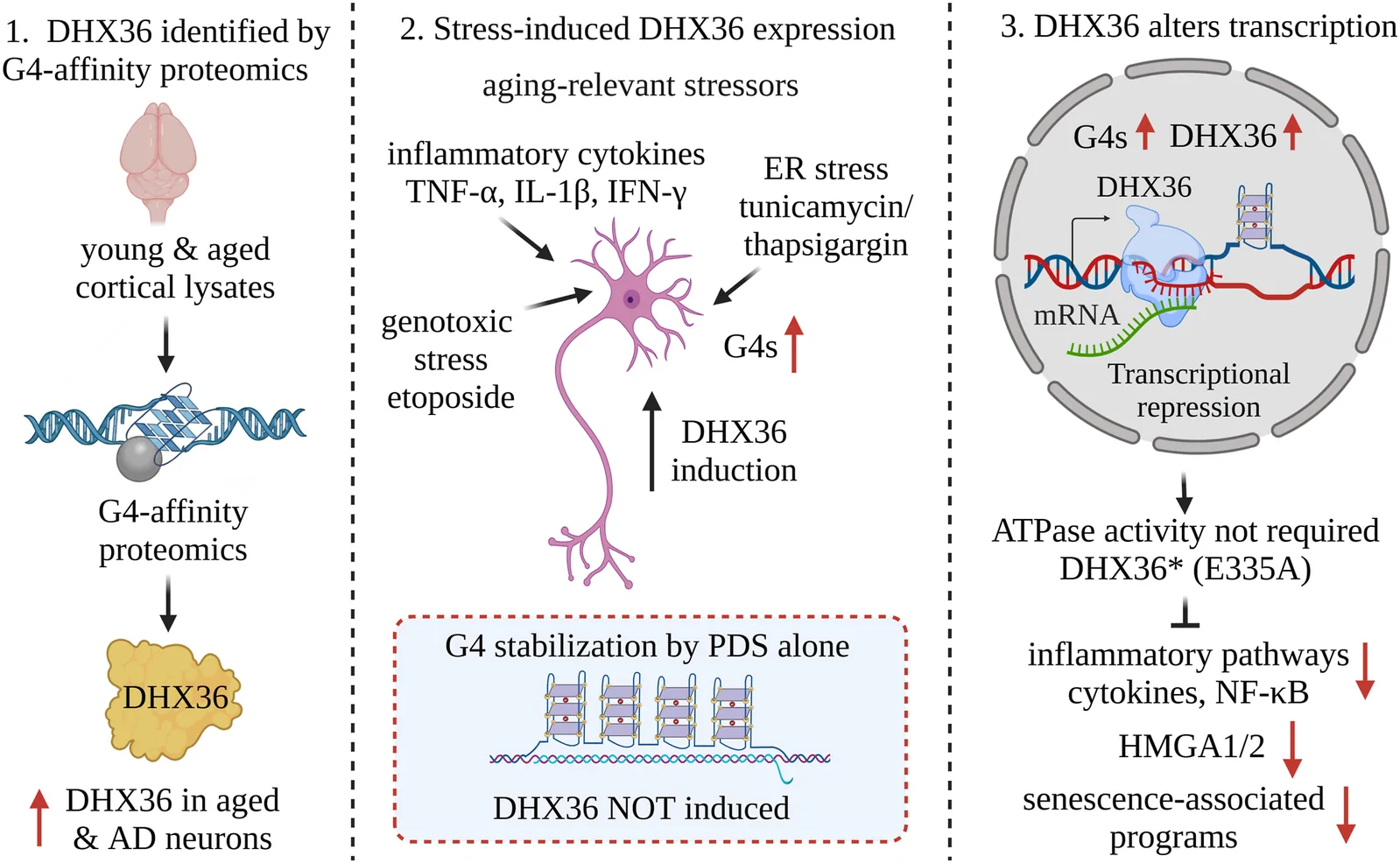
**Stress-dependent induction of DHX36 and its role in aging and Alzheimer’s disease.** (1) G4-affinity proteomics performed on young and aged cortical lysates identifies enrichment of the G4 helicase DHX36 in aged lysates, indicating increased association of DHX36 with G4 structures in the aging brain. (2) Exposure of neurons to aging-relevant stressors, including inflammatory cytokines (TNF-α, IL-1β, IFN-γ), endoplasmic reticulum (ER) stress (tunicamycin or thapsigargin), and genotoxic stress (etoposide), leads to increased G4 levels and robust induction of DHX36. In contrast, acute G4 stabilization by the G4 ligand PDS alone is insufficient to induce DHX36 expression. (3) Increased DHX36 alters neuronal transcription, suppressing pro-inflammatory signaling pathways and reducing expression of the chromatin architectural factors HMGA1 and HMGA2 and senescence-associated genes. These transcriptional effects do not require ATP-dependent unwinding.

The mechanism underlying the reduction of DHX36 by PDS remains to be defined. We analyzed the mouse *Dhx36* and human *DHX36* loci, including 2 kb upstream of the transcription start site, using G4Hunter and canonical G4 motif searches, and did not identify a canonical G4-forming motif on the sense strand. Our sequence analysis therefore does not support a simple model in which PDS represses *Dhx36* transcription by stabilizing a canonical G4 within the gene's sense strand, although it does not rule out noncanonical G4 structures or other indirect mechanisms. TOP2A activity contributes to DNA damage induced by both PDS and etoposide (70), yet these two compounds have opposite effects on DHX36 in our system. DHX36 regulation therefore cannot be explained by G4 stabilization or TOP2-associated DNA damage alone.

Increased DHX36 expression altered the neuronal transcriptome, with a predominant effect on gene suppression. RNA-sequencing analysis following DHX36 overexpression revealed broad modulation of multiple signaling pathways, including immune and inflammatory responses, stress and DNA damage pathways, apoptosis, cellular senescence, and aging (Fig. 10). Many of these pathways overlap with those activated during neuronal stress, suggesting that DHX36 feeds back to regulate stress-responsive gene networks that initially trigger its expression. An ATPase-deficient DHX36 mutant (E335A) reproduced the major transcriptional effects of wild-type DHX36, indicating that these effects do not require ATP-dependent unwinding. We had anticipated the opposite result, and it therefore warrants careful consideration. Non-catalytic functions are well documented across both DNA and RNA helicase families. An ATPase-deficient RECQL5 mutant binds RNA polymerase II and represses transcription as effectively as the wild-type protein (37, 38). Helicase-dead BLM retains adaptor activity in the regulation of c-Myc, promoting Fbw7-dependent ubiquitylation to the same extent as wild-type BLM (39). DDX5 and DDX17 regulate transcription through association with HDAC1 and other transcriptional regulators, a scaffolding function unrelated to duplex unwinding (32). Our observation, therefore, places DHX36 within an established pattern rather than in conflict with it.

Several mechanisms could account for a catalysis-independent transcriptional function of DHX36, and our data do not distinguish between them. First, DHX36 may act through G4 binding without unwinding. The DHX36-specific motif (DSM) in the N-terminus binds G4 structures with high affinity and lies outside the ATPase core, so E335A retains G4 recognition (40, 67, 68, 69, 71). Consistent with this, E335A has been used as a substrate-trapping variant to identify DHX36 target transcripts because it binds and does not release them (40). Second, DHX36 may act as a scaffold *via* protein-protein interactions independent of nucleic acid binding, as has been described for RECQL5 with RNA polymerase II (37). Third, DHX36 may influence chromatin state indirectly, for example, through the reduction of HMGA1 and HMGA2 that we observe here. Fourth, ectopic DHX36 may sequester G4 structures or G4-binding partners, altering their availability to other regulators. Our experiments do not determine which of these mechanisms operates, or whether the transcriptional activity depends on G4 binding, chromatin association, protein-protein interactions, or another non-catalytic function of DHX36. Two further observations bear on this interpretation. Loss of DHX36 increases NF-κB-regulated immune gene expression in an independent system (72). Knockout removes both DHX36 binding and catalysis, whereas E335A removes catalysis alone, which is consistent with a requirement for DHX36 engagement rather than turnover. Conversely, E335A behaves as a functional null in other contexts, including repeat-associated translation of the *C9orf72* expansion (73), indicating that the dependence on ATPase activity is process-specific rather than absolute. Distinguishing these possibilities will require a DSM-deleted, G4-binding-deficient mutant, direct mapping of DHX36 occupancy at target loci, and identification of its neuronal interaction partners.

We also found that DHX36 elevation led to downregulation of *MyD88*, a key adaptor in Toll-like receptor and interleukin signaling, along with suppression of multiple pro-inflammatory genes, suggesting an anti-inflammatory influence in neurons. In immune cells, DHX36 participates in nucleic acid-sensing pathways that activate NF-κB and interferon signaling (74). In contrast, our data indicate that increased DHX36 expression in neurons dampens excessive immune activation. These observations suggest that DHX36 function is cell type-dependent, promoting immune signaling in immune cells, whereas restraining inflammatory responses in neurons. This is consistent with observations in other systems, where loss of DHX36 leads to aberrant innate stress signaling (54, 75). In addition, DHX36 overexpression reduced the expression of genes associated with apoptosis and ferroptosis.

Our results also suggest a connection between DHX36 activity and chromatin state in aging neurons. We found that DHX36 overexpression downregulated HMGA1 and HMGA2, chromatin architectural proteins that maintain an open, transcriptionally permissive configuration by binding AT-rich DNA and displacing linker histone H1 (61, 62, 76). HMGA1/2 levels decline with organismal aging (77), consistent with reduced transcriptional plasticity. In the brain, decreased HMGA1/2 expression may contribute to age-associated epigenetic changes, including the accumulation of heterochromatin. The observed reduction of HMGA1/2 following DHX36 elevation, these findings suggest that DHX36 may regulate chromatin-associated mechanisms of the neuronal stress response during aging. Importantly, HMGA1 is an essential component of senescence-associated heterochromatin *foci* in aging cells, and its levels can regulate chromatin compartmentalization (78). Therefore, the DHX36-HMGA1/2 relationship may represent a link between persistent cellular stress and epigenetic remodeling in aging neurons.

Elevated DHX36 in Tau PS19 transgenic mice and human AD brains suggests that DHX36 is involved in the stress environment of neurodegenerative disease. We observed higher DHX36 levels in Tau PS19 brains than in wild-type controls, and similarly, AD patient brains showed increased DHX36 levels in neurons. Neurons in AD experience persistent stress signaling associated with tau and amyloid pathology, which may contribute to DHX36 regulation. We also observed differences in DHX36 expression between male and female brains in both mice and humans, consistent with reports of sex-dependent differences in immune responses, inflammatory signaling, and age-related chromatin remodeling (79). Determining whether DHX36 function differs by sex and whether it contributes to resilience or vulnerability in disease will require further investigation.

By demonstrating that DHX36 is induced by stressors that activate inflammatory, ER stress, or genotoxic signaling pathways, and that its elevation modulates inflammation, senescence-associated, and chromatin-linked pathways, our study identifies DHX36 as a mediator of neuronal adaptation in aging and neurodegeneration (Fig. 10). Several key questions remain. The upstream signaling pathways driving DHX36 induction, including potential sex-dependent mechanisms, warrant further investigation. The impact of sustained DHX36 elevation on chromatin organization, particularly through regulation of HMGA1/2, requires deeper study using chromatin accessibility and 3D genome approaches. Determining whether HMGA1 and HMGA2 are functionally required for specific downstream effects of DHX36 will require dedicated mechanistic studies. Manipulating DHX36 *in vivo* and assessing its effects on neuronal function and behavior will be important in future studies. Although our transcriptomic data suggest that DHX36 regulates defined gene networks, we did not directly map its binding to DNA or RNA at regulatory regions. Integration of DHX36 chromatin or RNA occupancy assays with G4 mapping and chromatin accessibility analyses will be important to distinguish direct from indirect effects. Defining the contribution of G4 binding to DHX36-mediated repression will also require a binding-deficient mutant. Moreover, because our findings are primarily based on a neuronal model and on mouse and human post-mortem tissue, *in vivo* studies will be essential to establish the functional role of DHX36 in aging and neurodegeneration. Taken together, these findings identify DHX36 as a stress-responsive regulator that links cellular stress to transcriptional control and neuroinflammatory signaling in neurons.

## Experimental procedures

### Chemicals and antibodies

Antibodies against DHX36 were from Novus Biologicals (catalog No: NB200-353); HMGA1 from ThermoFisher Scientific (catalog No: MA5-35485); HMGA2 from ThermoFisher Scientific (catalog No: PA5-21320); NeuN from ThermoFisher Scientific (catalog No: PA5-78499), and MAP2c from Novus Biologicals (catalog No: NB300-213). The anti-G-quadruplex antibody (clone BG4) was from EMD Millipore (catalog No: MABE917), and the anti-FLAG antibody used for BG4 detection was from Cell Signaling Technology (CST) (catalog No: 2368S). Anti-rabbit Alexa Fluor 488-labeled (catalog No: A11001), Anti-chicken Alexa Fluor 647-labeled (catalog No: A21449) secondary antibodies were from Life Technologies. All antibodies, with their sources, catalog numbers, applications, and working dilutions, are listed in Table 2. mScarlet, mScarlet-DHX36, and mScarlet-DHX36-E335A lentiviruses were custom-designed and procured from VectorBuilder.

**Table 2:** Antibodies used in this study

Antibody	Source	Catalog no.	Application	Dilution
DHX36	Novus Biologicals	NB200-353	Immunocytochemistry	1:500
DHX36	Novus Biologicals	NB200-353	Immunohistochemistry	1:500
DHX36	Novus Biologicals	NB200-353	Jess capillary immunoassay	1:100
HMGA1	ThermoFisher Scientific	MA5-35485	Immunocytochemistry	1:1000
HMGA1	ThermoFisher Scientific	MA5-35485	Immunohistochemistry	1:1000
HMGA1	ThermoFisher Scientific	MA5-35485	Jess capillary immunoassay	1:100
HMGA2	ThermoFisher Scientific	PA5-21320	Immunocytochemistry	1:1000
HMGA2	ThermoFisher Scientific	PA5-21320	Immunohistochemistry	1:1000
HMGA2	ThermoFisher Scientific	PA5-21320	Jess capillary immunoassay	1:100
NeuN	ThermoFisher Scientific	PA5-78499	Immunohistochemistry	1:500
MAP2c	Novus Biologicals	NB300-213	Immunocytochemistry	1:500
BG4 (anti-G-quadruplex)	MilliporeSigma	MABE917	Immunocytochemistry	1:50
Anti-FLAG	CST	2368S	Immunocytochemistry (BG4 detection)	1:500
Anti-rabbit Alexa Fluor 488	Life Technologies	A11001	Secondary (ICC/IHC)	1:1000
Anti-chicken Alexa Fluor 647	Life Technologies	A21449	Secondary (ICC/IHC)	1:1000

### DHX36 constructs

Lentiviral vectors encoding mScarlet, mScarlet-tagged wild-type human DHX36 (isoform 1, NM_020865.3), and mScarlet-tagged DHX36 carrying the E335A substitution were custom-designed and produced by VectorBuilder. The E335A substitution lies within the DEAH box (residues 334–337) of the RecA1 domain and abolishes ATP hydrolysis; it has previously been characterized as a catalytically inactive DHX36 variant. The DHX36-specific motif and the region required for G4-DNA and G4-RNA binding (residues 53–105) are unaffected by this substitution. All constructs were sequence-verified, and neurons were infected at matched multiplicity of infection.

### Animals

All experiments used wild-type C57BL/6 mice and Tau PS19 transgenic mice B6; C3-Tg (Prnp-MAPT∗P301S) PS19Vle/J and their non-transgenic littermates (The Jackson Laboratory, stock No. 008169), maintained on a 12-h light/dark cycle with food and water available *ad libitum*. Both male and female mice were used throughout, and sex is specified for each experiment. For G4-affinity proteomics, the frontal cortex was collected from young (3 months) and aged (26 months) male wild-type mice. For immunohistochemical analysis of DHX36 and HMGA1/A2 during aging, young (4 months) and aged (24 months) male and female wild-type mice were used. For analysis of DHX36 in tauopathy, aged (22–24 months) male and female Tau PS19 mice were compared with age-matched non-transgenic control littermates. Primary cortical neurons were prepared from wild-type C57BL/6 mouse embryos at E17–E18. Embryos of both sexes were pooled. All animal procedures, including breeding, aging, and transcardial perfusion, were approved by the Institutional Animal Care and Use Committee of the University of Texas Health Science Center at Houston under the protocol numbers AWC-26-0042 and AWC-24-0059.

### Isolation and culture of mouse primary neurons

Brain cortices from mouse embryos (E17-E18) were dissected, dissociated, and plated on 12-well tissue-culture plates (4 × 10^5^/well). Plates were coated with poly-D-lysine (Sigma-Aldrich; Catalog No: P6407) as described (41). Primary cortical neurons were grown in Neurobasal Plus Medium (Gibco; Catalog No: 21103049) supplemented with B-27 (Gibco; Catalog No: 17504001), GlutaMAX (Gibco; Catalog No: 35050061), and Penicillin-streptomycin (Gibco; Catalog No: 15140122). Neurons for imaging were plated on PDL-coated coverslips (Neuvitro; Catalog No: GG-12-PDL) on 24-well tissue-culture plates (3 × 10^5^/well). For RNA sequencing, three groups were prepared and processed in parallel: neurons infected with mScarlet, mScarlet-DHX36, or mScarlet-DHX36-E335A (viruses from VectorBuilder). The mScarlet lentivirus served as the control for both DHX36 constructs. The following day, the cells were lysed in 1 ml of RLT buffer (Qiagen), and the lysates were rapidly frozen and stored at −80 °C for RNA sequencing.

### Stress treatments in neurons

Primary cortical neurons were isolated from E17-E18 mouse embryos and maintained as described above. To examine how different stress modalities regulate Dhx36 expression and G4 formation, neurons at DIV14 were exposed to either pyridostatin (PDS) or to stressors that activate inflammatory, ER-stress, or genotoxic signaling pathways relevant to aging. For G4 stabilization, neurons were treated with PDS (2 μM; Sigma-Aldrich, SML2690-25MG) for 24 h, with DMSO used as the vehicle control. For signaling-driven stress conditions, neurons were treated for 24 h with pro-inflammatory cytokines TNF-α (10 ng/ml; Novus Biologicals, NBP2-35185), IL-1β (10 ng/ml; R&D Systems, 401-ML), or IFN-γ (10 ng/ml; R&D Systems, 485-MI), the ER stress inducers tunicamycin (1 μM; STEMCELL Technologies) or thapsigargin (1 μM; STEMCELL Technologies), or the DNA-damaging agent etoposide (5 μM; Sigma-Aldrich, E1383). All treatments were prepared in Neurobasal medium supplemented with B27, and matched vehicle controls were included for each condition. Following treatment, neurons were processed for immunocytochemistry, JESS capillary immunoassay, RT-qPCR, BG4, or N-TASQ staining as described in the corresponding sections.

### Sample preparation, library preparation, and mass spectrometry

Frontal cortex lysates from young (3-month-old) and aged (26-month-old) mice were incubated with SS-DNA oligos and G4-DNA forming oligos (ATG2700) immobilized on beads, followed by analysis using mass spectrometry. The brain lysates were lysed in 50 mM ammonium bicarbonate and 1 mM calcium chloride, and sonicated for 3 min at 30% amplitude, with 30-s pulses and 1-min rests at 4 °C. Protein concentrations were determined using the Bradford assay. For protein digestion, 25 μg of each lysate was digested with 0.5 μg of trypsin for 12 h at 37 °C. The concentration of tryptic peptides was measured using the Pierce Quantitative Peptide Assays kit (ThermoFisher Scientific, cat# 23275). A total of 25 μg of peptides was chromatographically separated using a custom-made reverse-phase C18 column within a pipette tip. The eluted peptides were fractionated into 15 distinct 150 μl fractions using a stepwise gradient of acetonitrile (2–35%) at pH 10. These fractions were then pooled into five groups (2 + 12 + 22%, 4 + 14 + 24%, 6 + 16 + 26%, 8 + 18 + 28%, and 10 + 20 + 35%) and desiccated under vacuum. The dried peptide fractions were reconstituted in a solution of 5% methanol and 0.1% formic acid in water for mass spectrometry analysis. Mass spectrometry was conducted using an Orbitrap Fusion mass spectrometer (Thermo Fisher Scientific) coupled with an Easy-nLC 1000 nanoflow LC system (Thermo Fisher Scientific). For peptide separation, an in-house trap column (2 cm × 100 μm) and a capillary column (5 cm × 150 μm), both packed with 1.9-μm Reprosil-Pur Basic C18 beads, were used. Separation was carried out with a 75-min discontinuous gradient of acetonitrile (4%–26%) and 0.1% formic acid at a flow rate of 800 nl/min. The instrument was operated in data-dependent mode using Xcalibur software version 4.1 (Thermo Fisher Scientific), which acquired fragmentation spectra for the top three most intense ions. The parent mass spectrum was recorded in the Orbitrap with a resolution of 120,000 over a 300 to 1400 m/z range. Ion fragmentation was performed using higher-energy collisional dissociation (HCD), and MS/MS spectra were collected in the ion trap in rapid scan mode. The MS/MS data were searched against the mouse RefSeq database (March 23, 2020 release, 62,232 entries) using the Mascot algorithm (Mascot 2.4, Matrix Science) within the Proteome Discoverer 2.1 interface. Search parameters included a precursor mass tolerance of 20 ppm and a fragment mass tolerance of 0.5 Da, with up to two missed tryptic cleavages allowed. Dynamic modifications, such as acetylation of the N-terminus and oxidation of methionine, were considered. Peptide Spectrum Matches (PSMs) were filtered at a 1% false discovery rate using Percolator validation based on q-value. PSMs from Proteome Discoverer 2.1 were processed with the 'gpGrouper' algorithm, which groups peptides at the gene level for MS1-based quantification across multiple gene products. Quantification of gene-protein products was performed using the label-free intensity-based absolute quantification (iBAQ) method. The resulting iBAQ values were normalized to the Fraction of Total (FOT), where FOT is calculated as the individual protein's iBAQ divided by the total iBAQ for all identified proteins in a given experiment. This normalization enables direct comparison of protein abundance across different samples.

### Sample preparation, library preparation, and mRNA sequencing

RNA was isolated from cell pellets using the RNeasy Mini (Qiagen) according to the manufacturer’s instructions with an elution volume of 30 μl. Sample QC was performed using RNA Screen Tape (Agilent Technologies Inc.) to assess the integrity of the RNA, and the RNA was quantified using Qubit Broad Range (Thermo Fisher). Library preparation was performed using the QIAseq Stranded mRNA Kit (Qiagen). From 100 ng of starting material, the mRNA was enriched, and the RNA was heat fragmented. After first and second strand synthesis, the cDNA was end-repaired and 3′ adenylated, and sequencing adapters were ligated to the overhangs. Adapted molecules were enriched using 16 cycles of PCR and purified using a bead-based cleanup method. Library preparation was quality-controlled using capillary electrophoresis (Agilent DNA 1000 Chip). High-quality libraries were pooled based on equimolar concentrations. The library pool(s) were quantified using quantitative PCR (qPCR), and the optimal concentration of the library pool was used to generate clusters on the surface of a flow cell before sequencing on a NextSeq (Illumina Inc.) instrument (2 × 75) according to the manufacturer's instructions. Raw data were demultiplexed, and FASTQ files for each sample were generated using the bcl2fastq software version 2.20.0.422 (Illumina Inc.).

### RNA-sequencing analysis

All primary analyses are carried out using CLC Genomics Server 23.0.5. The workflow “RNA-Seq Analysis” in CLC Genomics Server, using standard parameters, was employed to map reads to the mouse genome (GRCm38). The ‘Empirical analysis of DGE’ algorithm of the CLC Genomics Workbench 23.0.5 was used for differential expression analysis with default settings. It is an implementation of the ‘Exact Test’ for two-group comparisons, developed by Robinson and Smyth (1988) and incorporated into the EdgeR Bioconductor package (2009). Genes were considered differentially expressed at |log_2_ fold change| ≥ 0.6 and FDR < 0.05. For all unsupervised analyses, only mRNAs with at least 10 counts summed across all samples were considered. A variance stabilizing transformation was performed on the raw count matrix using the function *vst* of the R package DESeq2 version 1.28.1. The variance was calculated agnostically with respect to the predefined groups (blind = TRUE). The 35 genes with the highest variance across samples were selected for hierarchical clustering. The RNA-sequencing data were analyzed using iPathwayGuide, a tool provided by Adviata Bioinformatics. This analysis was performed within the context of pathways sourced from the KEGG (Kyoto Encyclopedia of Genes and Genomes) database, as well as gene ontologies obtained from the GO Consortium database. The analysis integrated these data sources to construct underlying topologies, which represent the network of genes and their directional interactions. These topologies were derived from the KEGG database utilizing iPathwayGuide, providing valuable insights into the biological pathways and processes influenced by the differential gene expression patterns identified through RNA sequencing. Overlap between the genes regulated by wild-type DHX36 and E335A was assessed using a hypergeometric test against the set of genes measured in both analyses, and the correspondence of fold changes was assessed using Pearson correlation.

### Immunocytochemistry

Cultured primary neurons from mouse embryos were grown on PDL-coated coverslips and infected with lentiviruses carrying either control mScarlet or mScarlet-DHX36. Neurons were washed with PBS and then fixed with a 4% paraformaldehyde-PBS solution for 15 min at room temperature. Cells were rinsed and incubated with a 5% bovine serum albumin-PBS and 0.3% Triton-X-100-PBS solution for 1 h at room temperature. Cells were incubated with primary antibodies DHX36, HMGA1, HMGA2, and MAP2c overnight at 4 °C. The next day, the cells were washed with PBS and incubated with secondary antibodies for 1 h at room temperature. Coverslips were mounted with a DAPI-containing mounting medium (ProLong Diamond Antifade Mountant with DAPI, catalog No. P36962, Invitrogen, Thermo Scientific), enabling visualization using a Nikon A1R Confocal Laser Scanning microscope.

### G4 detection using N-TASQ

G4 structures were visualized using the fluorescent probe N-TASQ following the same staining workflow as immunocytochemistry. Primary cortical neurons on PDL-coated coverslips were washed with PBS and fixed with 4% paraformaldehyde in PBS for 10 min at room temperature. After fixation, cells were permeabilized and blocked using 5% BSA in PBS containing 0.3% Triton X-100 for 1 h at room temperature. Coverslips were then incubated with 30 μM N-TASQ prepared in PBS containing 1% BSA for 30 min at room temperature in the dark. After incubation, cells were washed with PBS and mounted with DAPI-containing antifade medium (ProLong Diamond Antifade Mountant with DAPI, P36962, Invitrogen). All samples were imaged under identical laser, gain, and exposure settings using a Nikon A1R Confocal Laser Scanning Microscope, and nuclear G4 puncta were quantified using ImageJ.

### G4 detection using BG4 antibody

G4 structures were also detected with the BG4 antibody using the same fixation, permeabilization, and blocking workflow described above. Cells were incubated for 1 h at 37 °C with anti-G-quadruplex antibody (clone BG4, MilliporeSigma, MABE917) diluted 1:50 in PBS containing 1% BSA. Because BG4 carries a FLAG epitope tag, bound antibody was detected with an anti-FLAG antibody followed by a fluorophore-conjugated secondary antibody, each for 1 h at room temperature. Cells were co-stained for MAP2C, washed with PBS, and mounted with DAPI-containing antifade medium (ProLong Diamond Antifade Mountant with DAPI, P36962, Invitrogen). All samples were imaged under identical laser, gain, and exposure settings using a Nikon A1R Confocal Laser Scanning Microscope, and nuclear BG4 puncta were quantified in MAP2C-positive neurons using ImageJ.

### G4 prediction

Genomic sequences for mouse *Dhx36* (ENSMUSG00000027770) and human *DHX36* (ENSG00000174953) were retrieved from Ensembl with 2 kb of 5′ flanking sequence. G4-forming potential was assessed using G4Hunter (80) with a 25-nucleotide window, together with canonical G4 motif searching for 4 G ≥ 3 tracts separated by loops of one to seven nucleotides. Motifs falling within repeat-masked regions were excluded.

### Immunohistochemistry

Young (4–5 months) and aged (22–24 months) wild-type (C57BL/6) and Tau PS19 mice were transcardially perfused with PBS and fixed with 4% PFA. Brains were collected and stored in 30% sucrose until further use. 30 μm coronal brain sections were subjected to antigen-retrieval in sodium citrate buffer at 95 °C for 10 min. Sections were blocked in 5% normal goat serum, 1% bovine serum albumin, and 0.3% Triton-X-100 for 1 h at room temperature. Brain sections were incubated overnight at 4 °C with primary antibodies against DHX36, HMGA1, HMGA2, and NeuN. The next day, sections were washed with PBS and incubated with secondary antibodies for 1h at room temperature. Sections were mounted on a glass slide and covered with a DAPI-containing mounting medium (ProLong Diamond Antifade Mountant with DAPI, catalog No. P36962, Invitrogen, Thermo Scientific), allowing for visualization using a Nikon A1R Confocal Laser Scanning microscope.

### Human brain tissue

De-identified *postmortem* human hippocampal tissue from young normal, aged normal, and Alzheimer's disease cases was obtained from the BRAINS Research Lab brain biobank. Case demographics, including age, sex, post-mortem interval, and neuropathological staging, are provided in Table 1. Sections through the CA3 region were processed for immunohistochemistry as described above and stained for DHX36 and NeuN. For transcript and protein analysis, frozen hippocampal tissue was homogenized in RIPA lysis buffer. RNA was extracted, and *DHX36* transcript was measured by RT-qPCR with *TBP* as the reference gene, and DHX36 protein was measured by JESS capillary immunoassay with normalization to total protein, as described in the corresponding sections. All studies involving human tissue were conducted in accordance with the principles of the Declaration of Helsinki.

### Fluorescence microscopy and image analyses

Fixed cells were imaged using a Nikon A1R Confocal Laser Microscope, employing 20X, 40X, and 63X objectives. To ensure uniformity and comparability across all samples, imaging parameters, including light intensity, gain, and other relevant settings, were maintained constant throughout the imaging process. Subsequently, the acquired images were analyzed and quantified using ImageJ/Fiji software, enabling standardized measurements and evaluations of the experimental data.

### Capillary-based immunoassay *via* ProteinSimple

The capillary electrophoresis immunoassay was conducted using the JESS Automated Western Blot System (Catalog No. 004-650, ProteinSimple, Bio-Techne) to analyze total protein lysates from mouse neurons infected with either mScarlet or mScarlet-DHX36, and from human hippocampal tissue from young normal, aged normal, and Alzheimer’s disease cases. Lysates were extracted with RIPA lysis buffer (Catalog No. 20-188, EMD Millipore) supplemented with a Protease and Phosphatase Inhibitor Cocktail (Catalog No. 5872, Cell Signaling Technology) and then centrifuged at 12,000 rpm for 10 min at 4 °C. Total protein concentration was quantified using a BCA assay, and samples were subsequently diluted to 1 μg/μl in sample buffer (ProteinSimple, Bio-Techne). A master mix was prepared at a 4:1 protein-to-mix ratio, incorporating a 40 mM fluorescent molecular weight marker and dithiothreitol. Prior to loading, samples were denatured at 95 °C for 5 min. Proteins were separated in 25-well capillary plates. DHX36 was resolved using the 12 to 230 kDa separation module, and HMGA1 and HMGA2 were resolved using the 2 to 40 kDa separation module. Antibodies were diluted in antibody diluent (ProteinSimple, Bio-Techne) at the following concentrations: DHX36 (1:100), HMGA1 (1:100), and HMGA2 (1:100). Detection was performed using an HRP-conjugated anti-rabbit secondary antibody. For data analysis, relative protein levels were quantified by measuring the areas under the peaks in chemiluminescence chromatograms using the Compass for SW software (version 6.3.0, ProteinSimple, Bio-Techne). Protein levels were expressed as peak intensity, normalized to the total protein loading control.

### Real-time qPCR

Primary neurons were infected with lentiviruses carrying mScarlet, mScarlet-DHX36, or mScarlet-mutant DHX36 (E335A). Cells were lysed in RLT buffer obtained from Qiagen. Total RNA was extracted using the RNeasy Mini kit (Qiagen; catalog No: 74104). 1 μg of RNA was used to prepare the cDNA using iScript Reverse Transcription SuperMix (Bio-Rad; catalog No: 1708840), according to the manufacturer’s protocol. Real-time qPCR was performed using a Bio-Rad CFX96 machine with SSoAdvanced Universal SYBR Green (Bio-Rad; catalog no. 1725275) for visualization and quantification, according to the manufacturer’s instructions. Primer sequences used were, DHX36: FP: 5′-GTCTTTCTACCAGGCTGGGACA-3′, RP: 5′-GTGTCTGGTTGACGGTAGGCAT-3′; HMGA1: FP: 5′-AGTGCCAACTCCGAAGAGACCT-3′, RP: 5′-TTGGTTTCCTCCCTGGAGCTGT-3′; HMGA2: FP: 5′- AGAGGAAGACCCAAAGGCAGCA-3′, RP: 5′-GAGCAGGCTTCTTCTGAACGAC-3′. For validation of RNA-sequencing targets in neurons expressing mScarlet, mScarlet-DHX36, or mScarlet-DHX36-E335A, the following primers were used: Cxcl1: FP: 5′-TCCAGAGCTTGAAGGTGTTGCC-3′, RP: 5′-AACCAAGGGAGCTTCAGGGTCA-3′; Casp4: FP: 5′-GTGGTGAAAGAGGAGCTTACAGC 3′, RP: 5′-GCACCAGGAATGTGCTGTCTGA-3′; Il19: FP: 5′-CTGTGGACATGCGCCTCATAGA-3′, RP: 5′-TGAGGTTCTCCAGGGACAGGAT-3′; Tlr2: FP: 5′-ACAGCAAGGTCTTCCTGGTTCC-3′, RP: 5′-GCTCCCTTACAGGCTGAGTTCT-3′; Bcl3: FP: 5′-AGCAGTCGTCTCAGCTCCAATG-3′, RP: 5′ AGGCAGGTGTAGATGTTGTGGG-3′; Serpine1: FP: 5′-CCTCTTCCACAAGTCTGATGGC-3′, RP: 5′-GCAGTTCCACAACGTCATACTCG-3′; Cd14: FP: 5′ TTGAACCTCCGCAACGTGTCGT-3′, RP: 5′-CGCAGGAAAAGTTGAGCGAGTG-3′; Myd88: FP: 5′-ACCTGTGTCTGGTCCATTGCCA-3′, RP: 5′-GCTGAGTGCAAACTTGGTCTGG-3′; Pagr1a: FP: 5′-GAGCCAATGACACCAAAGGACTC-3′, RP: 5′-TGAGAGGACCTTATCCAGACGC-3′; Gpr17: FP: 5′-CGAAGACCTCTCTATGCTCACC-3′, RP: 5′-CGGTACAGTTGCAGGCAGACAA-3′. PCR conditions used were: 95 °C for 3 min, followed by 40 cycles of 95 °C for 10 s, and 60 °C for 30 s. TATA-binding protein (TBP) mRNA levels were used as an internal control for the target mRNAs. Normalization and relative fold change expression levels of all genes were calculated from the average threshold cycle number using the delta-delta Ct method.

### Statistical analysis

Image quantification was performed in ImageJ/Fiji, and statistical analyses in GraphPad Prism. In every experiment, *n* denotes the number of independent biological replicates, individual animals for tissue experiments, independent neuronal culture preparations for *in vitro* experiments, and individual cases for human tissue, and is stated in each figure legend along with the number of cells analyzed. Data are presented as mean ± SEM, and individual biological replicate values are shown as points on all bar graphs. Two-group comparisons were made using unpaired two-tailed Student's *t* tests. Comparisons across more than two groups were made using one-way ANOVA followed by Tukey's multiple comparisons test, except for the G4-DNA and ssDNA pulldown data in Figure 1*B*, which were analyzed using two-way ANOVA. The test applied, and the significance thresholds are stated in each figure legend; *p* < 0.05 was considered significant. Statistical analysis of differential gene expression is described in the RNA sequencing section.

### Ethical approval

All experimental protocols and procedures underwent approval by the University of Texas, McGovern Medical School at Houston, and the research was conducted in full compliance with the approved guidelines, ensuring adherence to ethical and scientific standards throughout the study. All animal procedures, including breeding, aging, and transcardial perfusion of wild-type and PS19 mice, were approved by the Institutional Animal Care and Use Committee of the University of Texas Health Science Center at Houston (Animal protocol numbers – AWC-26-0042 and AWC-24-0059). De-identified *postmortem* human hippocampal tissue from young normal, aged normal, and Alzheimer's disease cases was obtained from the BRAINS Research Lab brain biobank, and its use was approved by the Institutional Review Board of the University of Texas Health Science Center at Houston.

## Data availability

The RNA sequencing data generated in this study have been deposited in the NCBI Gene Expression Omnibus (GEO) under the accession number GSE344742 and will be publicly released upon publication. All remaining data are contained within the manuscript and supporting information. Any additional information is available upon request from Andrey S. Tsvetkov (andrey.s.tsvetkov@uth.tmc.edu).

## Supporting information

This article contains supporting information.

## Conflict of interest

The authors declare that they have no conflicts of interest with the contents of this article.
