# Supplementary material for: Stress-induced helicase DHX36 controls neuroinflammation
Source: J Biol Chem. 2026 Sep 23;302(11):113584. doi: 10.1016/j.jbc.2026.113584 (PMC13737755; doi:10.1016/j.jbc.2026.113584)
Supplement: Supplementary file 1 [file mmc1.pdf]

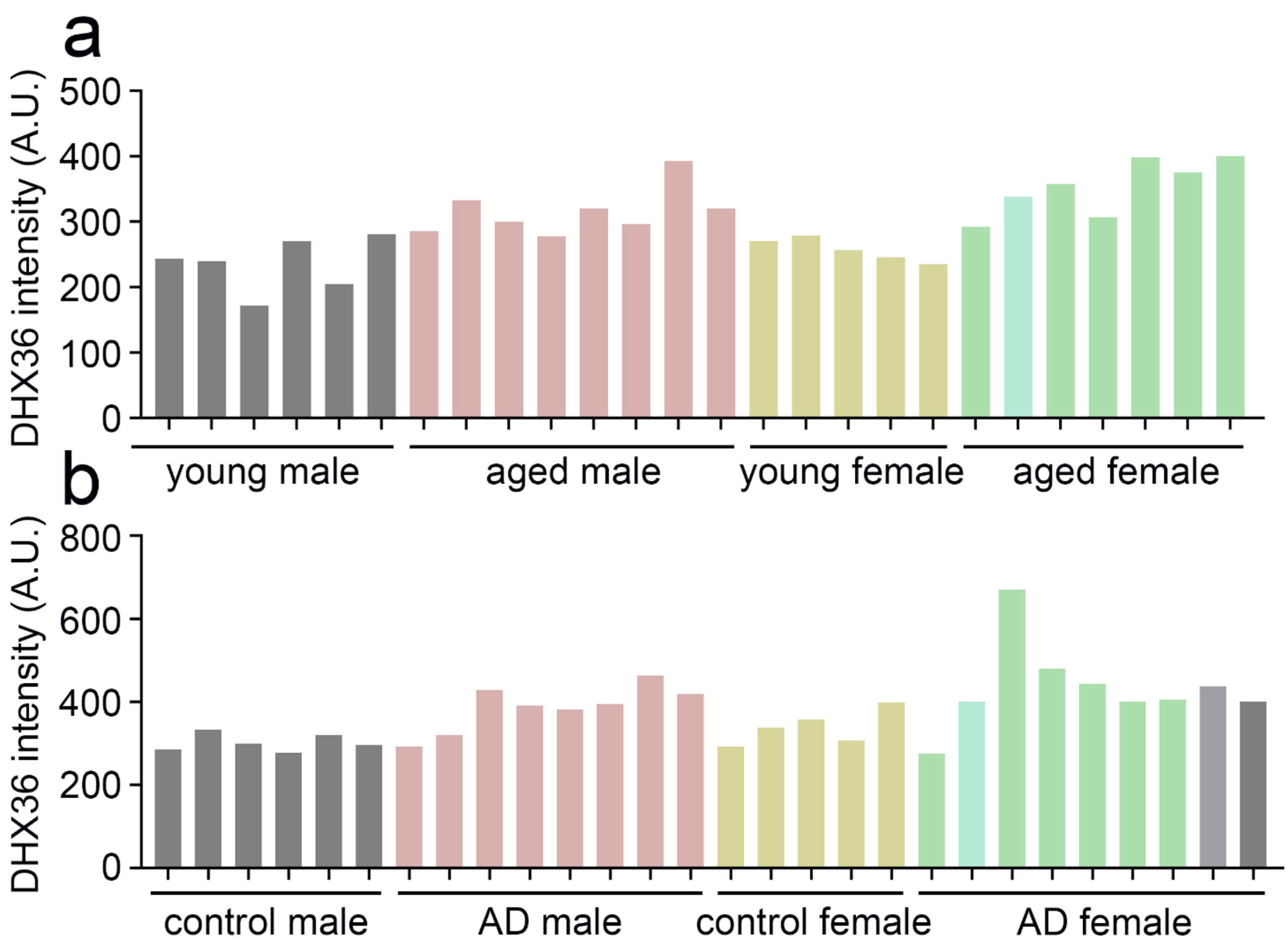

Figure S1. DHX36 intensity in all individual human hippocampal CA3 samples. (a) Individual DHX36 fluorescence intensity values measured from young male, aged male, young female, and aged female human hippocampal CA3 samples. Each bar represents one biological sample. These data illustrate the overall trend of increased DHX36 levels with aging in both males and females. **(b)** Individual DHX36 intensity values from control male, AD male, control female, and AD female CA3 samples. The plot illustrates the upward trend in DHX36 expression in AD cases compared to sex-matched controls. These plots display raw individual sample values and are intended to show only distribution and directional trends. No statistical comparisons were performed for this figure.

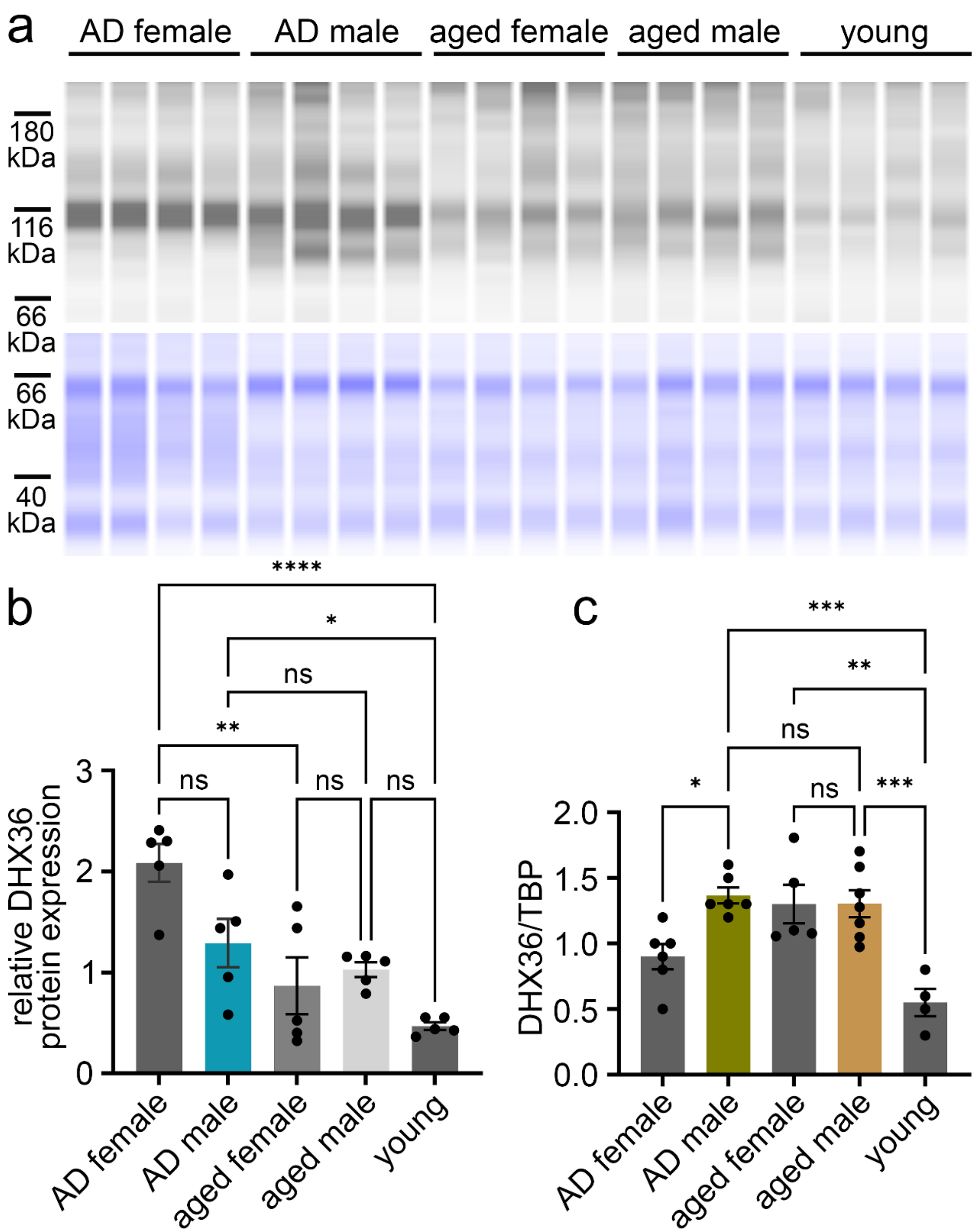

Figure S2. DHX36 protein and transcript levels are increased in the human brain of aged individuals and in Alzheimer's disease. (**a**) Representative Jess capillary immunoassay of DHX36 in human hippocampal lysates from young controls, aged controls, and Alzheimer's disease (AD) cases, separated by sex. DHX36 was detected at 116 kDa (upper panel); total protein is shown below as the loading control. (**b**) Quantification of DHX36 protein levels normalized to

total protein. DHX36 was significantly elevated in AD female and AD male cases relative to young controls, and in aged female cases relative to young controls. No significant differences were detected between male and female cases within the AD or aged groups. (**c**) *DHX36* transcript levels measured by RT-qPCR and normalized to *TBP*. *DHX36* was significantly increased in AD and aged cases relative to young controls, with no significant differences between males and females within groups. Data represent mean ± SEM. N = 5 per group (mentioned in Table 1). *p < 0.05, **p < 0.01, ***p < 0.001, ****p < 0.0001, one-way ANOVA with Tukey multiple comparison test.

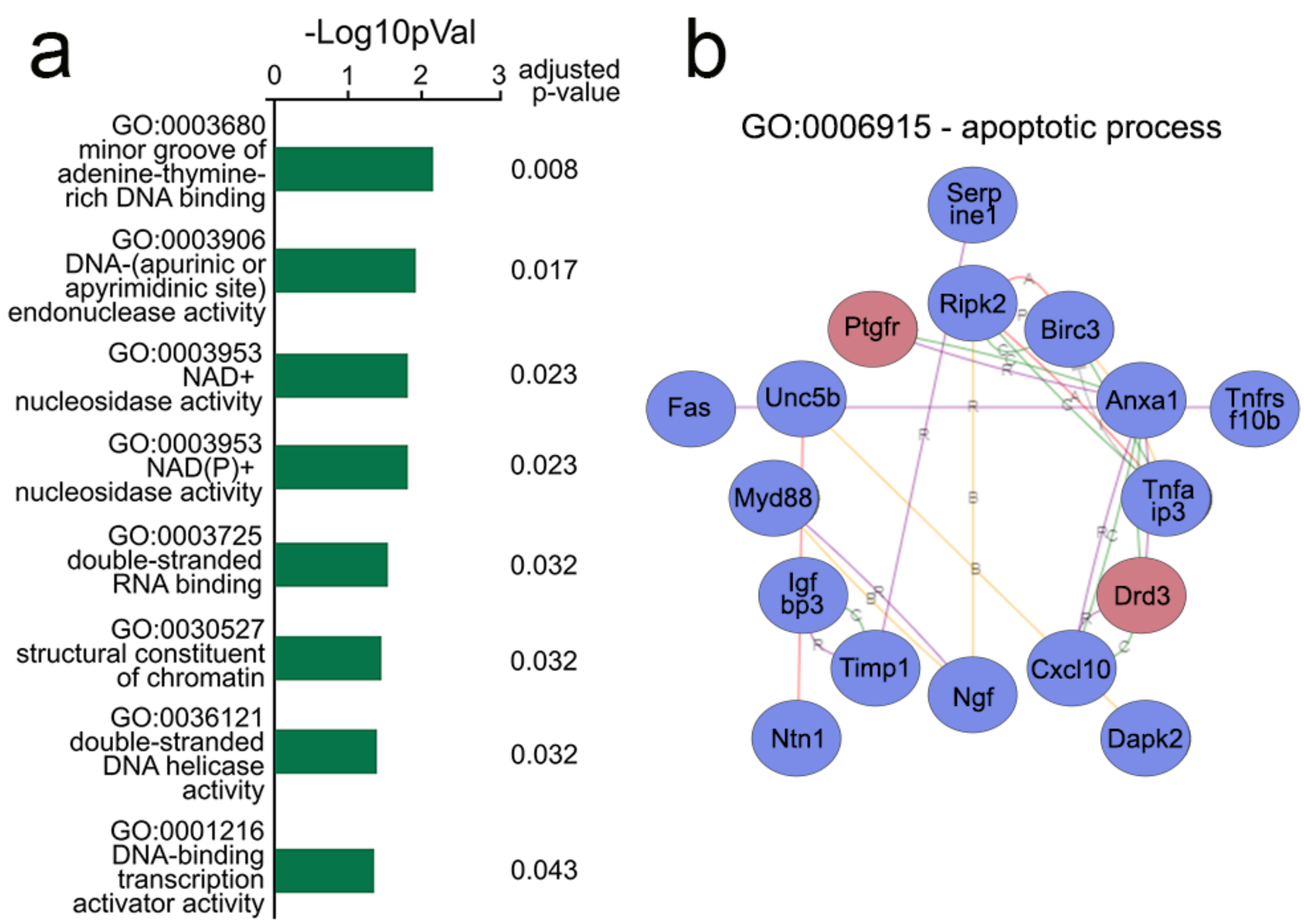

Figure S3. DHX36 influences chromatin-linked molecular functions and apoptotic gene networks. (a) Gene Ontology (GO) molecular function enrichment analysis of DHX36-regulated genes identifies significant enrichment in pathways associated with chromatin structure and nucleic acid interactions, including minor groove DNA binding, apurinic/apyrimidinic endonuclease activity, NAD^+/$NAD(P)^+$ nucleosidase activity, double-stranded RNA binding, structural chromatin components, and double-stranded DNA helicase activity. **(b)** Network visualization of DEGs associated with the GO term *apoptotic process* (GO:0006915), highlighting

DHX36-regulated pro- and anti-apoptotic genes such as *Fas*, *Ripk2*, *Birc3*, *Anxa1*, *Tnfαip3*, *Drd3*, *Ptgfr*, *Timp1*, *Cxcl10*, *Dapk2*, *Unc5b*, and *Ntn1*.

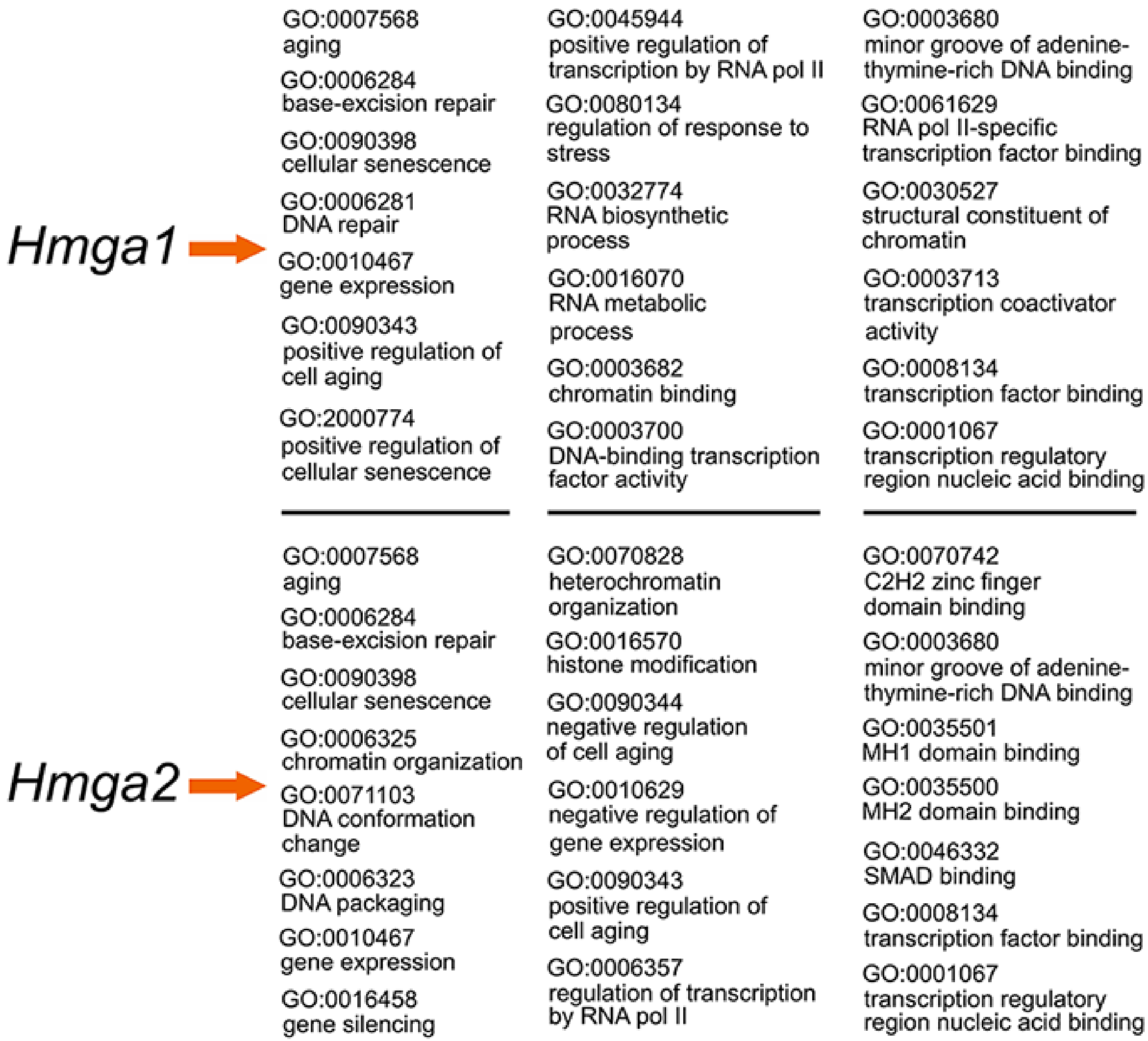

Figure S4. GO functional categories associated with Hmga1 and Hmga2 highlight their roles in chromatin regulation and cellular senescence. Hmga1-associated GO terms include aging (GO:0007568), base-excision repair (GO:0006284), cellular senescence (GO:0090398), DNA repair (GO:0006281), gene expression (GO:0010467), positive regulation of cell aging (GO:0090343), and positive regulation of cellular senescence (GO:2000774). Additional functional terms include regulation of transcription by RNA polymerase II, stress response, RNA biosynthetic and metabolic processes, chromatin binding, transcription factor activity, and DNA-binding transcription factor activity. **Hmga2-associated GO terms** include aging (GO:0007568),

base-excision repair (GO:0006284), cellular senescence (GO:0090398), chromatin organization (GO:0060325), DNA conformation change, DNA packaging, gene expression (GO:0010467), and gene silencing. Other terms encompass heterochromatin organization, histone modification, negative regulation of gene expression, positive regulation of cell aging, transcription by RNA polymerase II, and protein–DNA interaction categories, including C2H2 zinc finger binding, MH1/MH2 domain binding, SMAD binding, minor groove DNA binding, transcription factor binding, and nucleic acid binding within transcriptional regulatory regions.

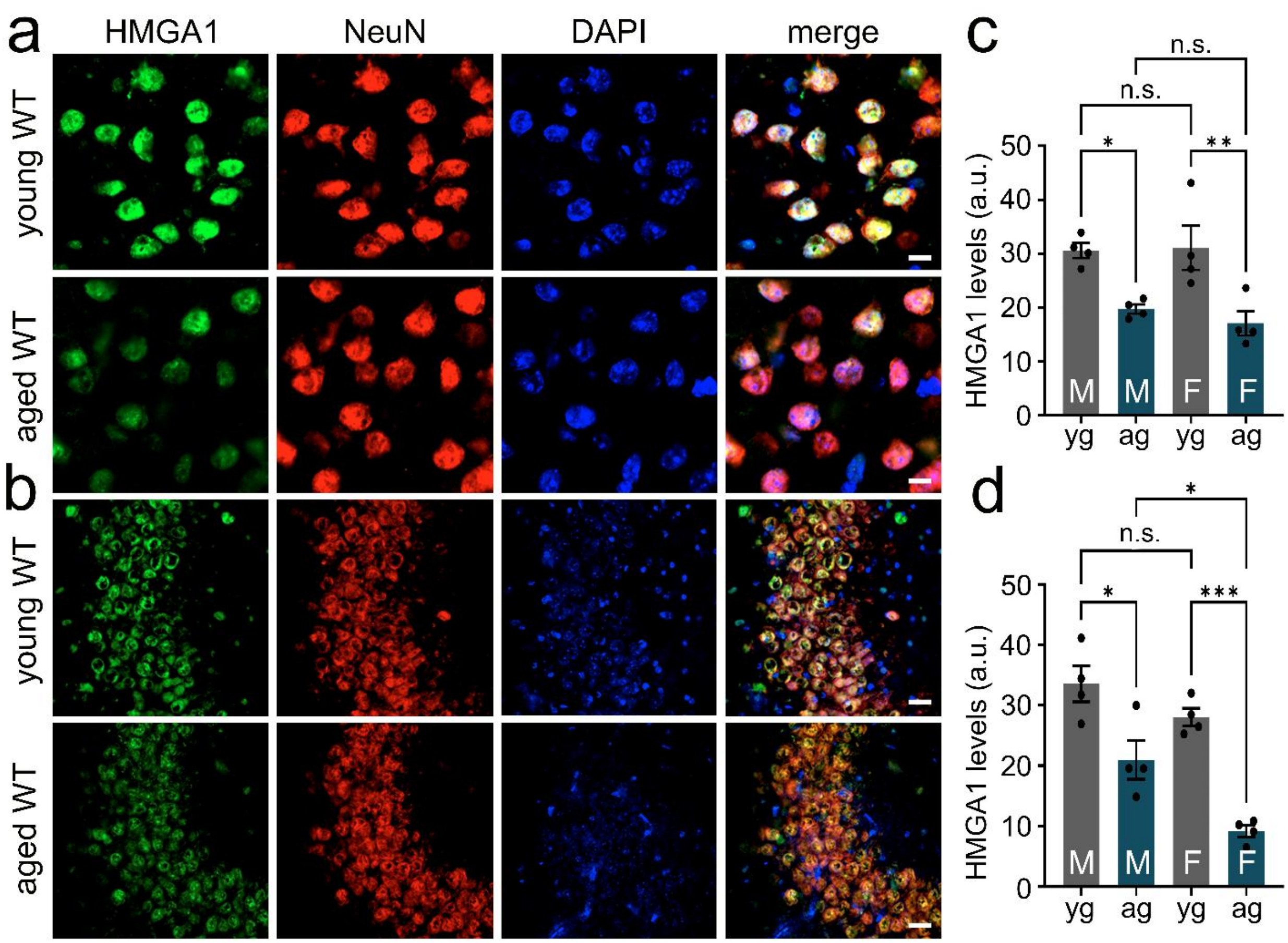

Figure S5. HMGA1 expression is reduced in the aged cortex and hippocampus. (a) Representative confocal images of coronal cortical sections from young and aged wild-type mice stained for HMGA1 (green), NeuN (red), and DAPI (blue). **(b)** Representative hippocampal sections from young and aged wild-type mice stained for HMGA1, NeuN, and DAPI. **(c)** Quantification of HMGA1 fluorescence intensity in cortical neurons reveals a significant decrease in aged males and females compared to their young counterparts. **(d)** Quantification of HMGA1 levels in hippocampal neurons reveals a robust reduction in aged mice compared to young controls

in both sexes. Fluorescence intensities were measured in ImageJ and normalized. Scale bar = 10 μm. N = 4 biological replicates per group (young male and female (4 months), aged male and female (24 months). Data represent mean ± SEM. Statistical comparisons were performed using one-way ANOVA. *p < 0.05, **p < 0.01, ***p < 0.001.

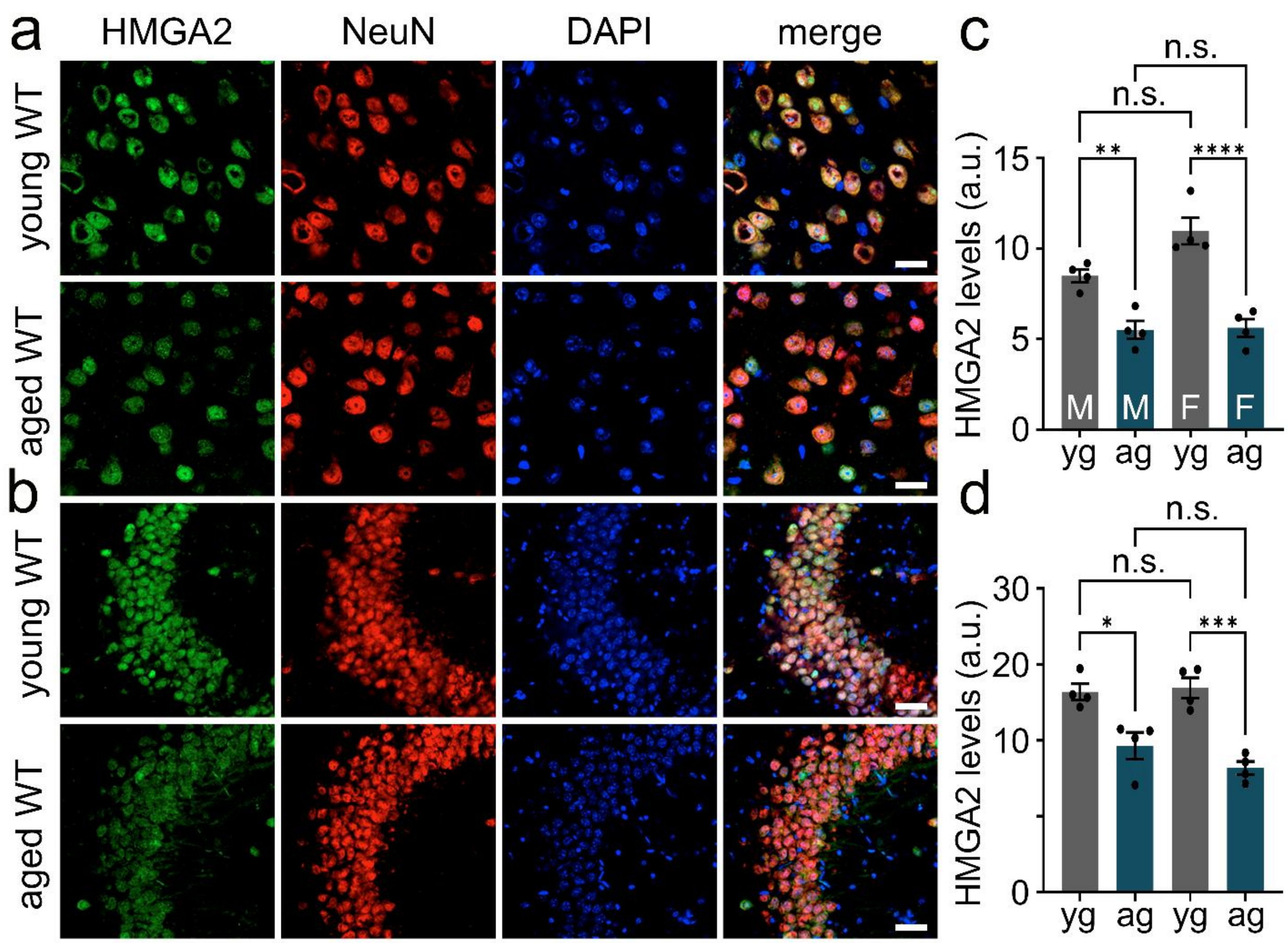

Figure S6. HMGA2 expression is reduced in the aged cortex and hippocampus. (a) Representative confocal images of coronal cortical sections from young and aged wild-type mice stained for HMGA2 (green), NeuN (red), and DAPI (blue). Aged cortex shows significantly reduced HMGA2 nuclear intensity compared to young controls. **(b)** Representative hippocampal CA3 sections from young and aged wild-type mice stained for HMGA2, NeuN, and DAPI. Aging leads to decreased HMGA2 staining in CA3 neurons. **(c)** Quantification of HMGA2 fluorescence intensity in cortical neurons reveals significantly lower HMGA2 levels in aged males and aged females compared to their young counterparts. **(d)** Quantification of HMGA2 intensity in hippocampal neurons also shows a significant reduction in aged mice compared with young animals, with no sex-specific differences. Fluorescence intensities were measured using ImageJ

and normalized. Scale bar = 10 µm. $N = 4$ biological replicates per group (young male and female (4 months), aged male and female (24 months). Data represent mean ± SEM. Statistical comparisons were performed using one-way ANOVA. *$p < 0.05$, **p < 0.01, ***$p < 0.001$, ****$p < 0.0001$.

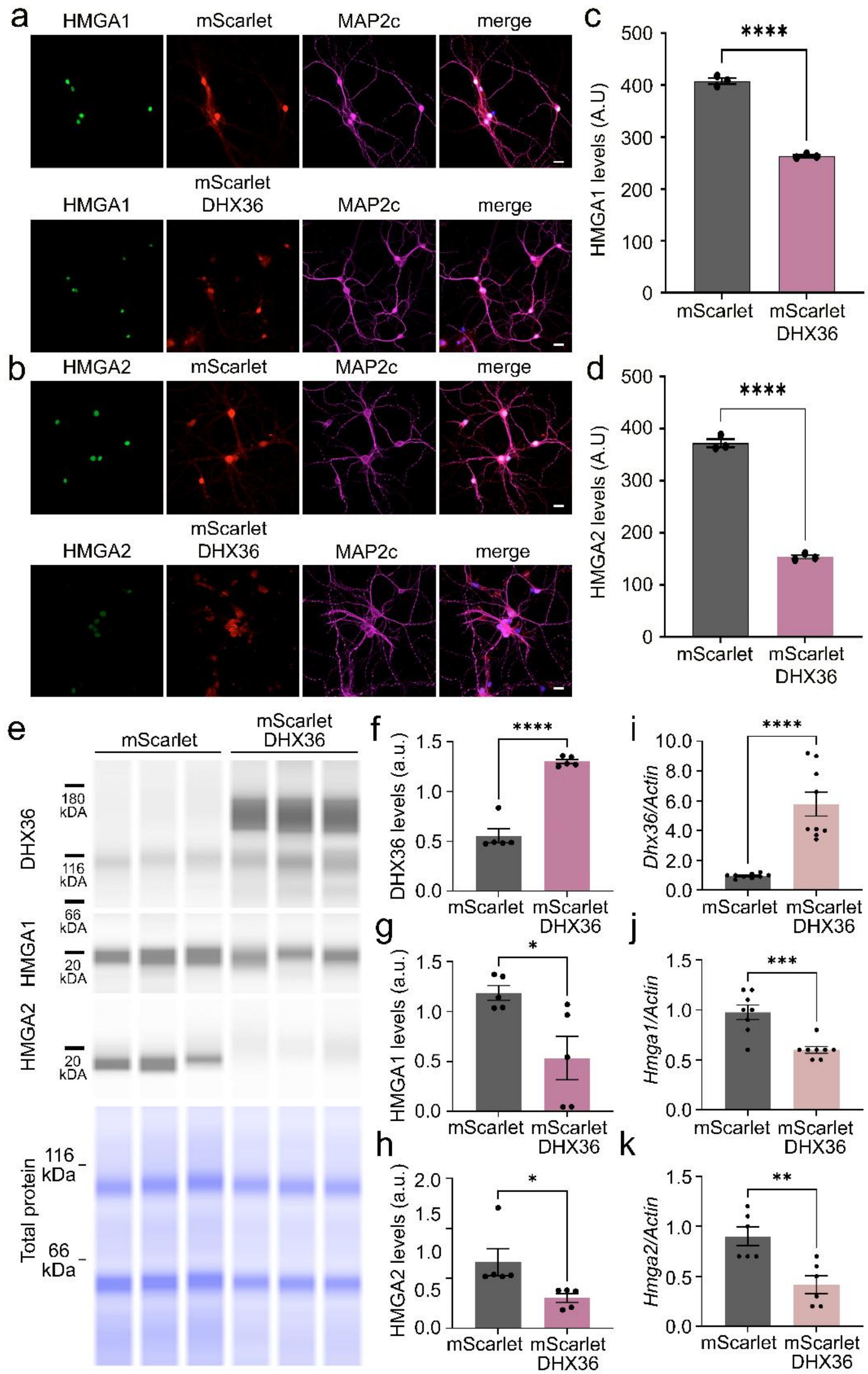

Figure S7. DHX36 overexpression suppresses HMGA1 and HMGA2 expression in neurons. **(a)** Representative confocal images of primary neurons (DIV14) infected with lentivirus expressing either mScarlet or mScarlet-DHX36. Cells were immunostained for HMGA1 (green), mScarlet (red), MAP2c (magenta), and DAPI (blue). DHX36 overexpression significantly reduced HMGA1 staining intensity in neuronal nuclei. **(b)** Representative confocal images stained for HMGA2 show a similar reduction in HMGA2 intensity in neurons overexpressing DHX36. **(c–d)** Quantification of HMGA1 **(c)** and HMGA2 **(d)** fluorescence intensity demonstrates a significant decrease in both proteins in DHX36-overexpressing neurons, N=3. **(e)** Representative **Jess capillary immunoassay** outputs for DHX36, HMGA1, and HMGA2 in neurons expressing mScarlet or mScarlet–DHX36, with total-protein normalization. **(f–h)** Quantification of Jess signal, DHX36 protein is significantly elevated in mScarlet-DHX36 neurons **(f)**, while HMGA1 **(g)** and HMGA2 **(h)** protein levels are significantly reduced, N=5. **(i–k)** qPCR analysis of *Dhx36* **(i)**, *Hmga1* **(j)**, and *Hmga2* **(k)** mRNA expression reveals robust transcriptional upregulation of *Dhx36* upon lentiviral infection and significant downregulation of both *Hmga1* and *Hmga2*. All values were normalized to *Actin*, N=8. Neurons were analyzed at DIV14. Data represent mean ± SEM. Statistical comparisons were performed using unpaired two-tailed t-tests. $^{*}p < 0.05$, $^{**}p < 0.01$, $^{***}p < 0.001$, $^{****}p < 0.0001$.

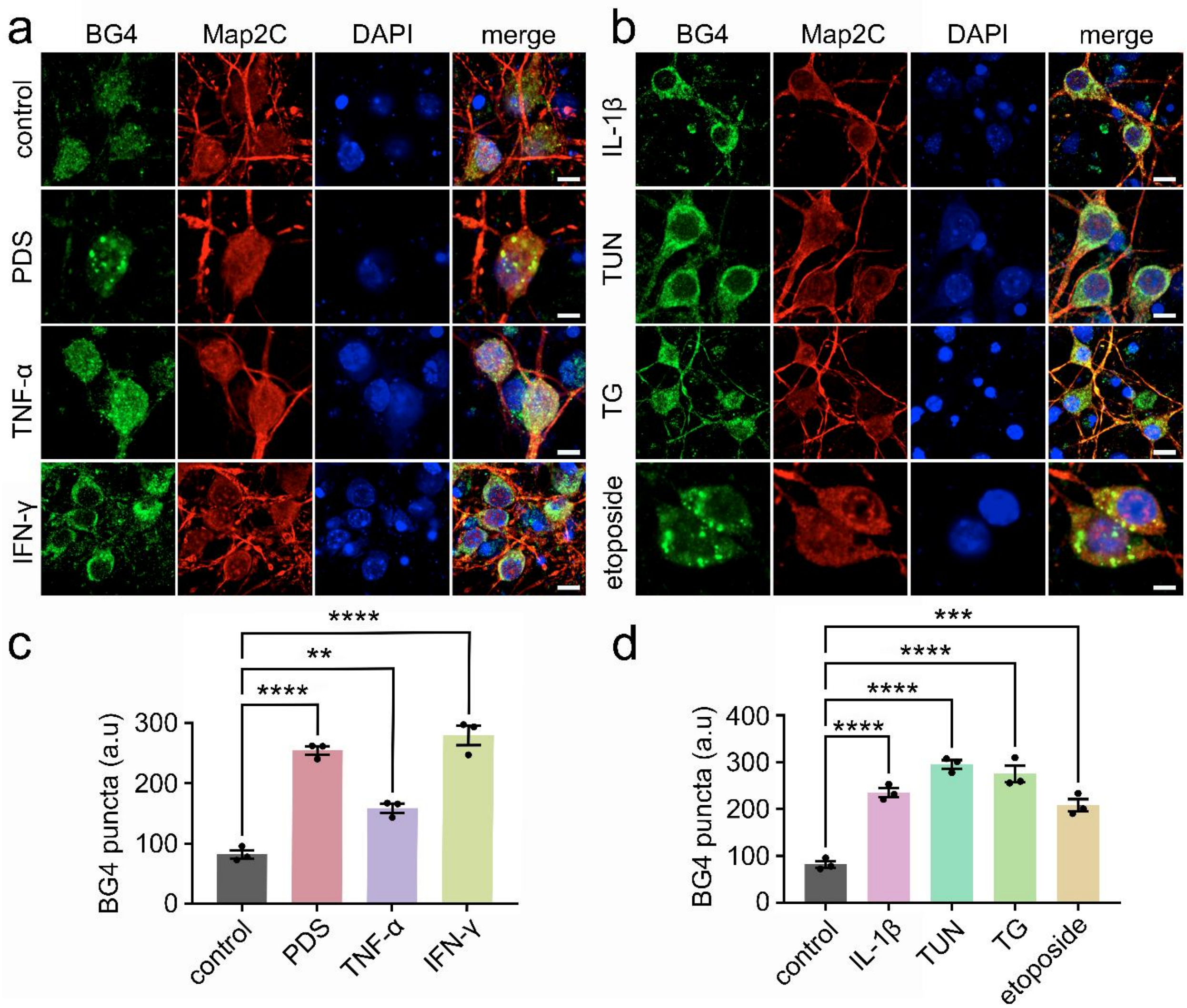

Figure S8. BG4 antibody staining confirms the stress-induced accumulation of G-quadruplexes in neurons. (**a**) Representative confocal images of primary mouse neurons treated with G4 stabilization (PDS, 2 μM) or inflammatory stressors (TNF-α, 10 ng/mL; IFN-γ, 10 ng/mL) and stained with the G-quadruplex antibody BG4 (green), Map2C (red), and DAPI (blue). All stress conditions increased BG4 fluorescence and puncta formation, indicating increased G4 puncta compared to controls. (**b**) Representative images of neurons treated with IL-1β (10 ng/mL), ER stressors tunicamycin (TUN, 1 μM) or thapsigargin (TG, 1 μM), or DNA-damage stress (etoposide, 5 μM). Each treatment enhanced the BG4 puncta signal, consistent with the increased formation of G4 structures. (**c**) Quantification of BG4 puncta intensity under PDS and inflammatory stress (TNF-α, IFN-γ) shows a robust increase in neuronal G4 formation. (**d**) Quantification of BG4 puncta intensity following IL-1β, ER stressors (TUN, TG), and etoposide shows a significant elevation in G4 levels. All treatments were applied for 24 h at DIV14 to model

PDS or cellular stress. BG4 detects G4 structures independently of N-TASQ, and both probes showed the same stress-associated increase (compare Figure 7). Data represent mean ± SEM. N = 3. ****p < 0.0001, one-way ANOVA with multiple comparisons. Scale bar = 10 μm.

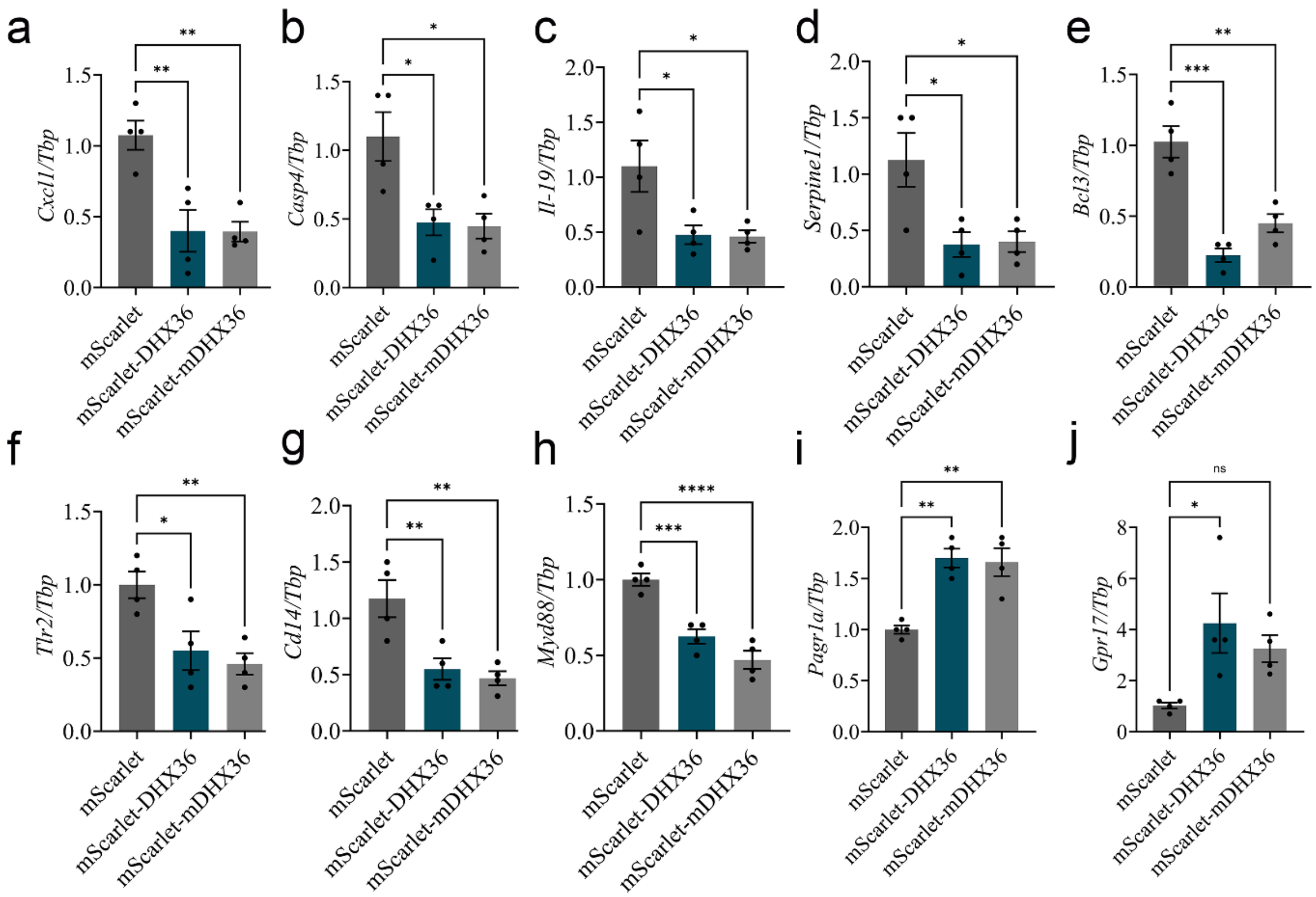

Figure S9. RT-qPCR validation of DHX36-regulated genes in neurons expressing wild-type or ATPase-deficient DHX36. Primary cortical neurons were infected at DIV14 with lentiviruses expressing mScarlet, mScarlet-DHX36, or mScarlet-mDHX36 (E335A ATPase-deficient mutant), and transcript levels of ten genes identified as differentially expressed by RNA sequencing were measured by RT-qPCR. (a–h) Genes downregulated by DHX36: *Cxcl1* (a), *Casp4* (b), *Il19* (c), *Serpine1* (d), *Bcl3* (e), *Tlr2* (f), *Cd14* (g), and *Myd88* (h). (i–j) Genes upregulated by DHX36: *Pagr1a* (i) and *Gpr17* (j). Wild-type DHX36 and the E335A mutant produced changes in the same direction and of comparable magnitude for all ten genes, consistent with the RNA sequencing data. All values were normalized to *Tbp*. Data represent mean ± SEM. N = 4. ns, not significant; *p < 0.05, **p < 0.01, ***p < 0.001, ****p < 0.0001, one-way ANOVA with Tukey multiple comparison test
